# The SCN9A channel and plasma membrane depolarization promote cellular senescence through Rb pathway

**DOI:** 10.1111/acel.12736

**Published:** 2018-02-15

**Authors:** Marine Warnier, Jean‐Michel Flaman, Christophe Chouabe, Clotilde Wiel, Baptiste Gras, Audrey Griveau, Elena Blanc, Jean‐Philippe Foy, Pauline Mathot, Pierre Saintigny, Fabien Van Coppenolle, David Vindrieux, Nadine Martin, David Bernard

**Affiliations:** ^1^ Inserm U1052, CNRS UMR 5286 Université de Lyon & Centre Léon Bérard, Centre de Recherche en Cancérologie de Lyon Lyon France; ^2^ Inserm UMR‐U1060 CarMeN Laboratory INRA U1235, INSA‐Lyon Facultés de médecine Rockefeller University Lyon 1 Lyon France

**Keywords:** oncogene, plasma membrane potential, Rb, SCN9A, senescence

## Abstract

Oncogenic signals lead to premature senescence in normal human cells causing a proliferation arrest and the elimination of these defective cells by immune cells. Oncogene‐induced senescence (OIS) prevents aberrant cell division and tumor initiation. In order to identify new regulators of OIS, we performed a loss‐of‐function genetic screen and identified that the loss of SCN9A allowed cells to escape from OIS. The expression of this sodium channel increased in senescent cells during OIS. This upregulation was mediated by NF‐κB transcription factors, which are well‐known regulators of senescence. Importantly, the induction of SCN9A by an oncogenic signal or by p53 activation led to plasma membrane depolarization, which in turn, was able to induce premature senescence. Computational and experimental analyses revealed that SCN9A and plasma membrane depolarization mediated the repression of mitotic genes through a calcium/Rb/E2F pathway to promote senescence. Taken together, our work delineates a new pathway, which involves the NF‐κB transcription factor, SCN9A expression, plasma membrane depolarization, increased calcium, the Rb/E2F pathway and mitotic gene repression in the regulation of senescence. This work thus provides new insight into the involvement of ion channels and plasma membrane potential in the control of senescence.

## 1 INTRODUCTION

A state of senescence is mainly characterized by a stable proliferation arrest, the acquisition of a senescence‐associated secretory programme (SASP), and a senescence‐associated β‐galactosidase activity (SA‐β‐Gal). Normal human cells enter into a state of senescence in response to numerous stresses, such as oncogenic signals, short telomeres, genotoxic or oxidative stresses. Senescence is a protective mechanism against tumor development, as it avoids cell division and it allows the elimination of potentially harmful cells by the immune system, through the SASP (Adams, 2009; Collado & Serrano, 2010; Kang et al., 2011).

Oncogenic signals have been shown to promote senescence in normal human cells by regulating different pathways, mainly centered on the DNA damage‐p53 pathway and/or the p16/Rb pathways. Nevertheless, a growing body of evidence supports the involvement of other pathways in the regulation of senescence, and in particular, in that of oncogene‐induced senescence (OIS) (Bianchi‐Smiraglia & Nikiforov, 2012; Christoffersen et al., 2010; Cipriano et al., 2011; Humbert et al., 2010; Lin et al., 2010; Scurr et al., 2010).

Ions and their channels are involved in most, if not all, the basic cell processes; nevertheless, their interests in cancer research in general and in OIS in particular have so far scarcely been studied. Ion channels control fluxes of numerous ions, such as calcium, which regulates a variety of cellular functions. Owing to their biological importance, as cofactors of numerous enzymes and as they are required for numerous functions, such as cytoskeleton movement and cell adherence, calcium channels and calcium have been extensively investigated (Becchetti & Arcangeli, 2010; Cuddapah & Sontheimer, 2011; Lee, Davis, Roberts‐Thomson & Monteith, 2011; Monteith, McAndrew, Faddy & Roberts‐Thomson, 2007; Prevarskaya, Ouadid‐Ahidouch, Skryma & Shuba, 2014; Prevarskaya, Skryma & Shuba, 2010). Other ion channel families, such as the ones controlling sodium or potassium, have been thoroughly studied in the context of action potential, signal transmission and in the biology of excitable cells. However, their functions in nonexcitable cells, in controlling cellular senescence and in participating in cancer cell biology, remain largely uninvestigated.

Here, based on an unbiased loss‐of‐function genetic approach, we identified, among several new senescence regulators, SCN9A (or Nav1.7), a sodium channel known to regulate plasma membrane depolarization. The mechanisms underlying NF‐κB/SCN9A/plasma membrane depolarization‐mediated OIS were unveiled, from the upstream regulation of SCN9A by NF‐κB following an oncogenic stress, to the involvement of SCN9A, plasma membrane depolarization, and calcium in the regulation of the Rb/E2F pathway, a key pathway regulating cell proliferation. Hence, our work sheds new light on a novel pathway controlling senescence.

## 2 RESULTS

### 2.1 SCN9A loss‐of‐function promotes escape from OIS

To identify new regulators of senescence, we performed a genetic screen using a shRNA library covering the whole genome in normal human fibroblasts induced to senescence by an oncogenic stress (Vindrieux et al., 2013). We thus obtained a list of genes inducing an escape from OIS when knocked down (Figure S1) and were particularly interested in the SCN9A sodium channel, which has so far never been associated with OIS. This plasma membrane channel belongs to the voltage‐gated sodium channel (Nav1), and it forms a sodium‐selective channel through which sodium ions pass according to their electrochemical gradient. The fact that this channel and its family have never been reported to regulate cellular senescence, prompted us to investigate the role of SCN9A in OIS.

**Figure 1:**
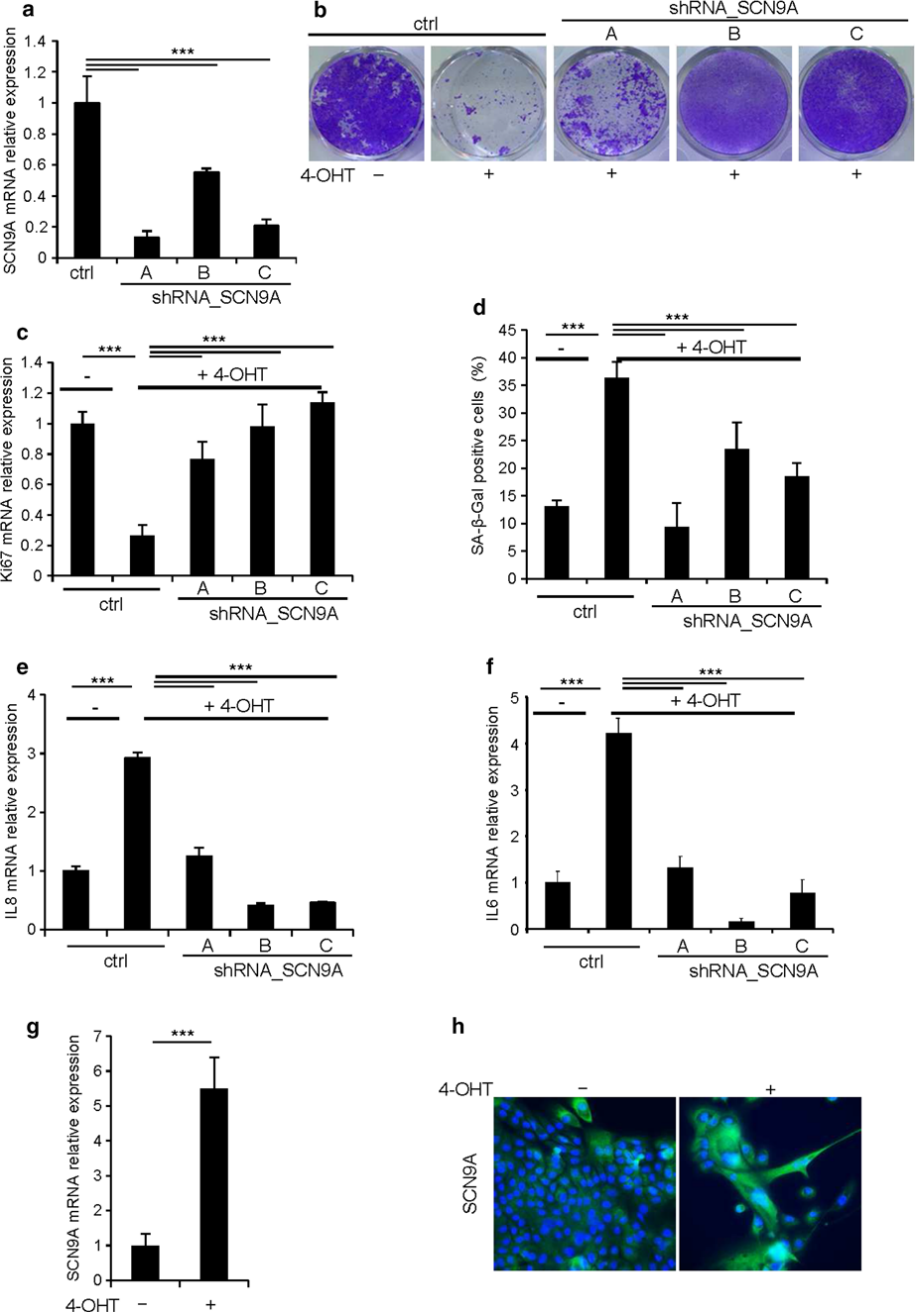
Loss of SCN9A enables cells to escape from oncogene‐induced senescence. HEC‐TM cells were infected with a control (ctrl) or with three different SCN9A‐targeting shRNA retroviral vectors (shRNA_SCN9A A, B, C) and selected using puromycin. (a) RNAs were prepared, reverse‐transcribed (RT), and the SCN9A transcripts were quantified by qPCR. Results were normalized with respect to the level of GAPDH transcript. (b–f) The day after seeding, cells were treated daily with 4‐OHT to activate MEK. Five days later, (b) cells were fixed and stained using crystal violet, (c) RNAs were prepared, reverse‐transcribed, and Ki67 transcripts were quantified by quantitative PCR, (d) cells were stained for SA‐β‐Gal activity and the number of SA‐β‐Gal‐positive cells was counted in each condition, and (e) IL8 and (f) IL6 were quantified by quantitative PCR. RT‐qPCR results were normalized against GAPDH mRNA levels. (g,h) After 5 d of treatment with 4‐OHT, (g) RNAs were prepared from HEC‐TM cell and analyzed for SCN9A expression by RT‐qPCR. The expression of SCN9A mRNA was normalized against GAPDH mRNA levels, (h) HEC‐TM cells were fixed, and SCN9A was detected by immunofluorescence. The nuclei were counterstained using Hoechst prior to microscopic visualization. The experiments shown are representative of at least three biological repeats. Statistical analysis was performed with the Student's *t* test, *** means *p* < .001

We next wanted to confirm and extend these first results obtained in human fibroblasts in epithelial cells, cells at the origin of carcinoma. We then used a model of OIS in human epithelial cells, a model we have extensively characterized (Lallet‐Daher et al., 2013; Wiel et al., 2014, 2016). These cells stably expressed both hTert to be immortalized and MEK:ER, a 4‐hydroxytamoxifen (4‐OHT) inducible oncogene (HEC‐TM cells), to induce the oncogenic signal. To assess the effect of SCN9A knockdown in HEC‐TM, we used three different SCN9A‐targeting shRNAs (Figures 1a and S2). SNC9A antibody specificity was validated by checking signal in SCN9A transfected cells (Figure S2a). As expected, inducing the oncogenic stress (+4‐OHT) resulted in proliferation arrest, as demonstrated by their reduced ability to form colonies (Figure 1b) and by the decrease in the level of the proliferation marker KI67 (Figure 1c), while it led to an increase in the SA‐β‐Gal activity (Figure 1d) and in the expression of two SASP components IL8 and IL6 (Figure 1e,f), both major hallmarks of senescence. Strikingly, the knockdown of SCN9A in HEC‐TM cells overcame all of the hallmarks of senescence induced by an oncogenic stress (Figure 1b–f), suggesting that SCN9A loss‐of‐function promotes escape from OIS. Having functionally demonstrated that SCN9A impacts OIS, we wondered whether its expression may be regulated by an oncogenic stress. Interestingly, we observed that the induction of an oncogenic stress (+4‐OHT) in HEC‐TM resulted in increased SCN9A mRNA (Figure 1g) and protein levels, as clearly evidenced by immunofluorescence (Figure 1h).

### 2.2 NF‐κB transcription factors mediate the induction of SCN9A during OIS

**Figure 2:**
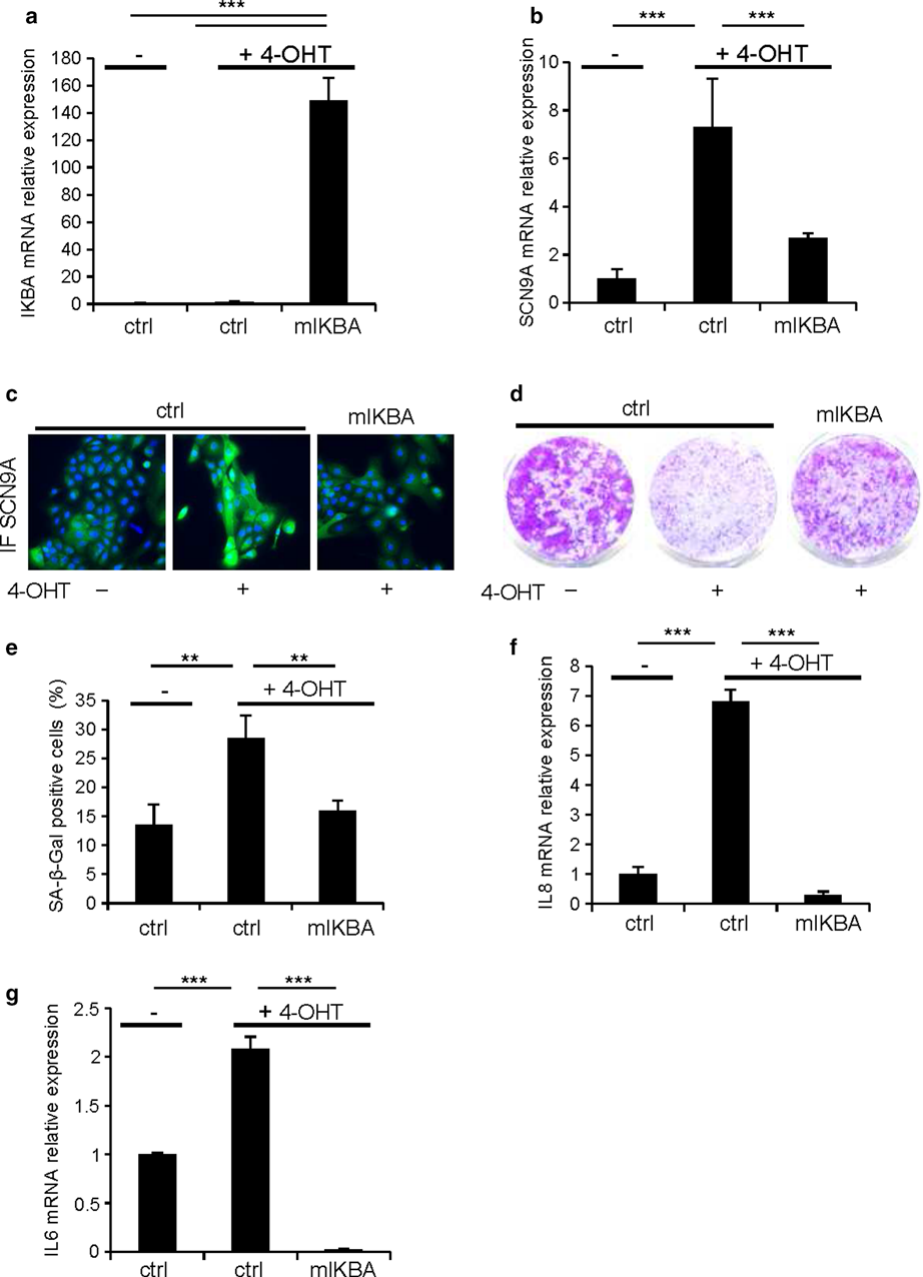
NF‐κB mediates increased SCN9A expression and oncogene‐induced senescence. (a,b) HEC‐TM cells were infected with control or mIKBA‐expressing retroviral vectors and puromycin selected. The day after seeding, they were treated daily with 4‐OHT for 4 d and RNAs were prepared, and (a) mIKBA expression and (b) SCN9A expression were measured by RT‐qPCR. Their relative mRNA expressions were normalized against GAPDH mRNA levels. (c) Immunofluorescence staining against SCN9A was performed. (d) Cells were fixed and stained using crystal violet. (e) Cells were fixed and stained for SA‐β‐Gal activity. (f,g) RNAs were prepared, and the mRNA levels of (f) IL8 and (g) IL6 were analyzed by RT‐qPCR. Their expressions were normalized against GAPDH mRNA. The experiments shown are representative of at least two biological repeats. Statistical analysis was performed with the Student's *t* test, ** means *p* < .01, ***p < .001

To unravel the mechanisms underlying the upregulation of SCN9A following an oncogenic stress, we attempted to identify factors upstream of SCN9A, which may be implicated in its OIS‐mediated regulation. We particularly focused on NF‐κB transcription factors, as they were reported to induce SCN9A expression in neurons (Huang et al., 2014) and are well‐known regulators of senescence (Acosta et al., 2008; Bernard et al., 2004; Chien et al., 2011; Ferrand et al., 2015). We examined whether or not the inhibition of NF‐κB transcription factors, either by constitutively expressing a stabilized version of IKBA (mIKBA), a well‐known inhibitor of NF‐κB, or by knocking down the expression of RELA (Figures 2a and S3a), the main subunit of the NF‐κB transcription factors, blocked the induction of SCN9A during OIS. Both approaches significantly reduced the induction of SCN9A following an oncogenic stress at the mRNA (Figures 2b and S3b), as well as at the protein (Figures 2c and S3c) levels. Furthermore, inhibition of NF‐κB transcription factors was able to induce an escape from OIS similar to that observed after SCN9A knockdown (Figure 1), as evidenced in colony‐forming assays (Figures 2d and S3d), in SA‐β‐Gal activity assays (Figures 2e and S3e), and in the mRNA levels of IL6/IL8 SASP components (Figures 2f,g, and S3f,g), thus supporting the functional relevance of our observations. These data support the view that the induction of SCN9A following an oncogenic stress is mediated by NF‐κB transcription factors and that this induction is required for OIS.

### 2.3 Oncogenic stress/NF‐κB‐mediated SCN9A induction causes plasma membrane depolarization

**Figure 3:**
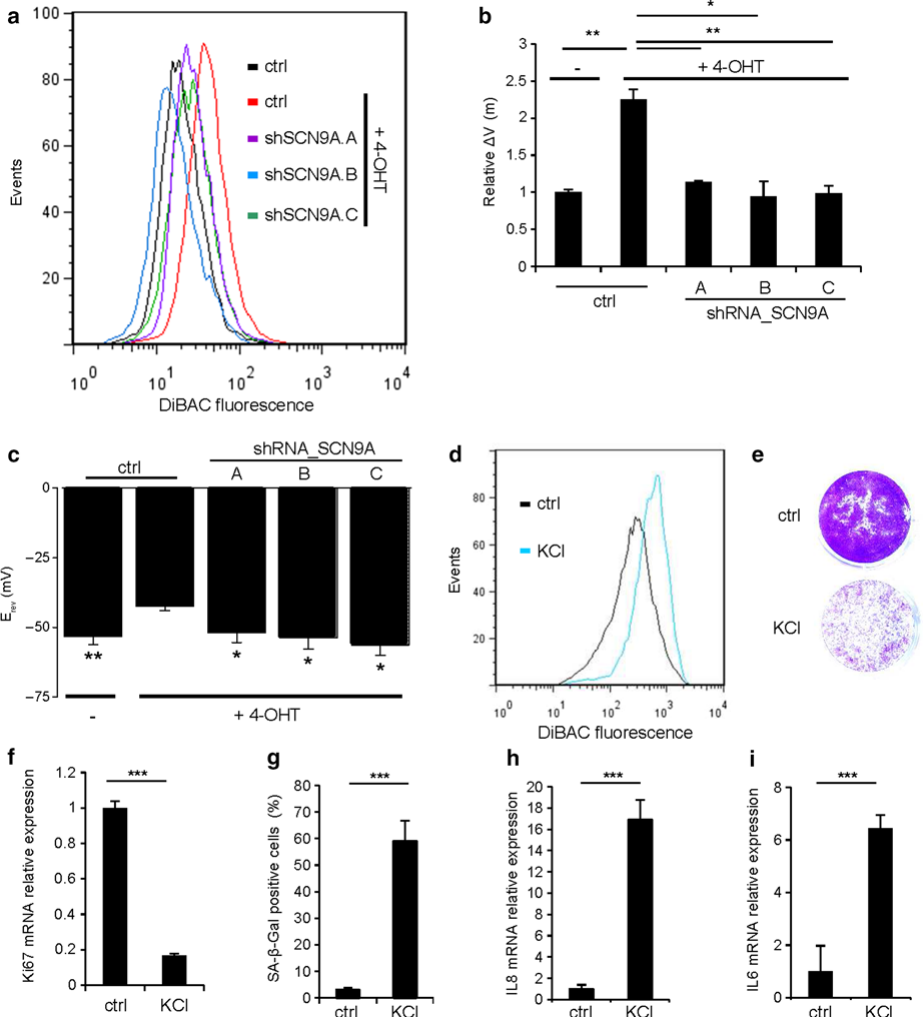
Increase in SCN9A during oncogene‐induced senescence leads to plasma membrane depolarization. (a,b) HEC‐TM cells were infected with control or SCN9A‐shRNA‐encoding vectors, selected, and treated daily with or without 4‐OHT for 4 d. They were then incubated with the fluorescent DiBAC4 dye to measure their relative plasma membrane potential (ΔV(m)) by flow cytometry with 10,000 events. (a) The fluorescence intensity profile shown is representative of three experiments. (b) Histograms showing the mean relative DiBAC4 fluorescence from three experiments are shown. (c) Histograms showing the mean reversal potentials (Erev) of ramp membrane currents determined by the patch‐clamp technique in the different indicated conditions (9 to 11 cells patched in each condition). (d) HEC‐TM cells were incubated for 6 d in normal medium or in a medium containing 65 mM KCl to induce plasma membrane depolarization. Plasma membrane depolarization was confirmed by flow cytometry using the DiBAC4 fluorescent probe. (e) Cells were fixed and stained using crystal violet. (f) RNAs were prepared and RT‐qPCR performed against Ki67 proliferation marker. Results were normalized against GAPDH mRNA levels. (g) Cells were fixed and assayed for their SA‐β‐Gal activity. Percentage of SA‐β‐Gal‐positive cells is displayed. (h,i) RNAs were prepared, and (h) IL8 or (i) IL6 expression was analyzed by RT‐qPCR. Expressions were normalized against GAPDH mRNA levels. The experiments shown are representative of three repeats. Statistical analysis was performed with the Student's *t* test, * means *p* < .05, **p < .01, ***p < .001

The SCN9A sodium channel, if active, allows entry of sodium into the cells and then promotes plasma membrane depolarization. To assess whether the increase in SCN9A levels induced by an oncogenic stress and by NF‐κB transcription factors resulted in changes in the plasma membrane potential, we conducted two different approaches. We first evaluated by flow cytometry the uptake by cells of the DiBAC fluorescent dye, which increases proportionally to plasma membrane depolarization (and thus decreases in hyperpolarized cells). Interestingly, senescent cells (+4‐OHT) displayed an increase in the intensity of the fluorescent signal (Figures 3a,b, and S4a,b), while the same treatment in SCN9A knockdown cells resulted in a decreased plasma membrane depolarization (Figure 3a,b), similar to that observed in NF‐κB inhibited cells (Figure S4a,b). To confirm these results, our second approach was based on an electrophysiological study. We observed a plasma membrane depolarization in senescent cells, and this depolarization was lost in SCN9A knockdown cells (Figure 3c). To conclude this part, senescent cells are depolarized and SCN9A is mediating this depolarization.

### 2.4 Plasma membrane depolarization provokes premature senescence

As SCN9A mediates plasma membrane depolarization induced by the oncogenic stress, we next wanted to know whether this plasma membrane depolarization can participate to senescence induction. To test this hypothesis, we treated HEC‐TM with a well‐established plasma membrane depolarizer, namely KCl. Interestingly, forced plasma membrane depolarization (Figure 3d) induced premature senescence, as it resulted in proliferation arrest (Figure 3e,f), increased SA‐β‐Gal activity (Figure 3g), and increased mRNA levels of SASP components (Figure 3h,i). KCl‐induced senescence was not impacted by knocking down SCN9A and was able to prevent OIS escape induced by SCN9A knockdown (Figure S5). Overall, these last findings suggest that induction of the plasma membrane depolarization, downstream of a NF‐κB/SCN9A pathway, participates in the regulation of OIS.

### 2.5 Plasma membrane depolarization, as oncogenic stress, represses mitotic gene expression

**Figure 4:**
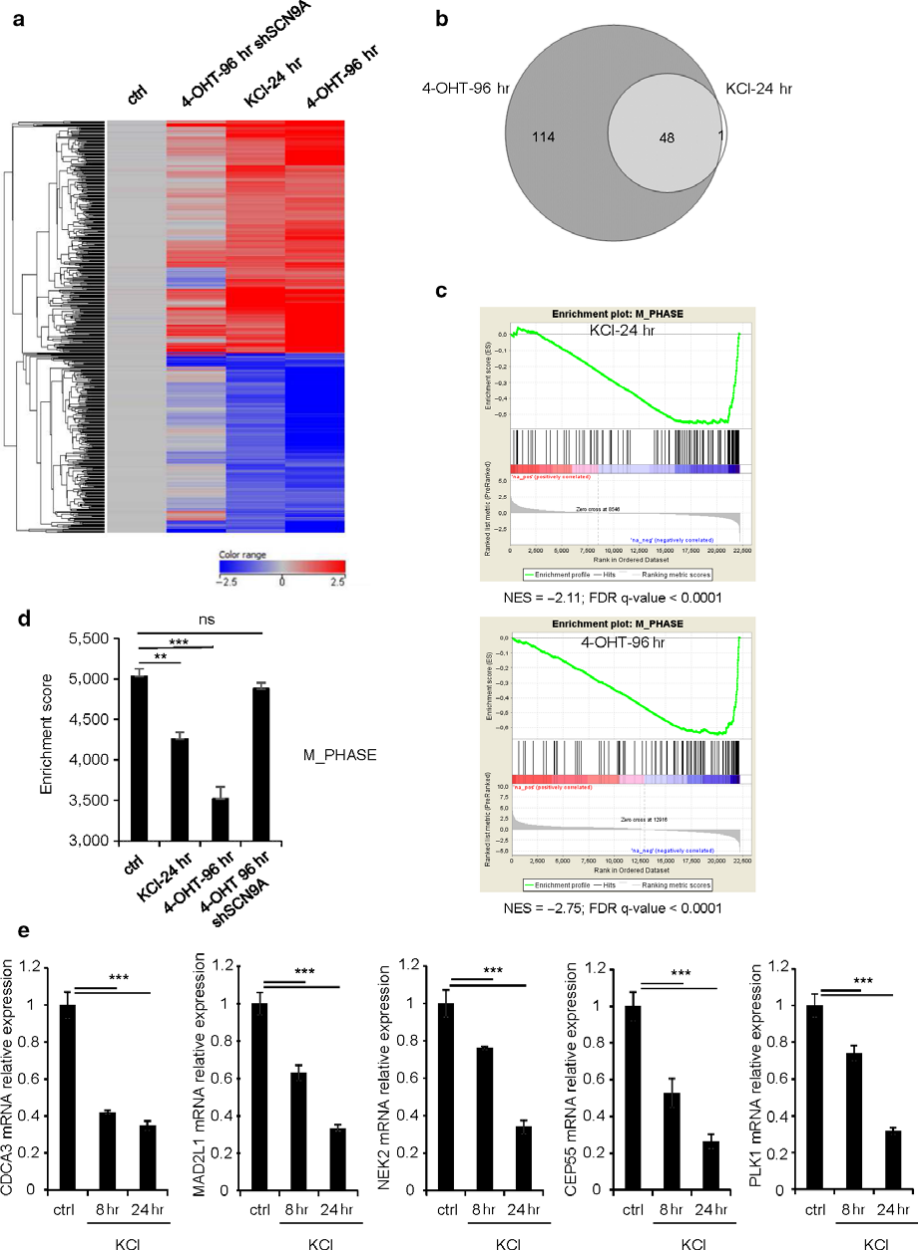
Plasma membrane depolarization, like oncogenic stress, turns off mitotic genes. (a) Heatmap representation showing the expression pattern of 532 differentially expressed genes shared by HEC‐TM cells treated either with KCl during 24 hr (KCl‐24hr) or with 4‐OHT during 96 hr (4‐OHT‐96 hr) compared to untreated HEC‐TM cells (Ctrl), and HEC‐TM transduced with a shRNA against SCNA9 treated with 4‐OHT for 96 hr (4‐OHT‐96 hr shSCN9A). Differentially expressed genes for KCl‐24 hr and 4‐OHT‐96 hr were identified from Agilent microarray data using unpaired *t* test analysis with Bonferroni multiple testing correction with fold change ≥2.0 and a *p* value cutoff of <.01. Common regulated genes were selected following a Venn diagram analysis. Pseudocolors indicate differential expression compared to HEC‐TM untreated cells sample (ctrl) (blue indicates downregulated genes and red upregulated transcripts). This dendrogram shows the hierarchical clustering (unsupervised; Pearson centered correlation with centroid linkage rules). (b) Venn diagram comparing the gene sets enriched for downregulated genes in KCl‐24 hr and 4‐OHT‐96 hr conditions according to GSEA (FDR <5%). (c) A gene set enrichment analysis (GSEA) was performed for 1,039 GO gene sets using the log2‐transformed fold change of expression between control and KCl‐24 hr cells as well as between controls and 4‐OHT‐96 hr cells (see Methods). The “Phase M” pathway (from GO) was one of the most significant gene sets that were downregulated in KCl‐24 hr and 4‐OHT‐96 hr compared to control samples, respectively. (d) The enrichment score of the “Phase_M” pathway from Gene Ontology was computed in each sample, using the ssGSEA tool (see Method). The scores were statistically compared between the three replicates of the different experimental conditions: control, KCl‐24 hr, 4‐OHT‐96 hr, and 4‐OHT‐96 hr shSCN9A. (e) Cells were treated with 65 mM KCl for 0, 8, or 24 hr before RNA extraction. RT‐qPCR was then performed on the indicated genes, and their levels of expression were normalized against GAPDH mRNA levels. This experiment was performed three times. Statistical analysis was performed with the Student's *t* test, ** means *p* < .01, ***p < .001

In order to define the mechanisms by which plasma membrane depolarization contributes to OIS, we performed transcriptome analysis of nontreated, 4‐OHT or KCl‐treated HEC‐TM cells, to identify common pathways between OIS and plasma membrane depolarization‐induced senescence and being reverted by the knockdown of SCN9A. Hierarchical clustering revealed a large number of genes similarly regulated in OIS‐ and KCl‐induced senescence, a regulation which was reverted upon SCN9A knockdown (Figure 4a). Gene Ontology (GO) analysis highlighted numerous shared pathways among the downregulated genes (Table S1), but none among the upregulated genes with a corrected *p* value <1.00E‐7. Strikingly, GSEA revealed that almost every single enriched pathway in the downregulated genes in KCl‐treated cells overlapped with enriched pathways in the downregulated genes during OIS (Figure 4b). By focusing on the GO results for downregulated genes, we found that pathways covering mitosis were highly enriched in OIS cells, as expected, as well as in KCl‐treated after 24 hr, suggesting that plasma membrane depolarization might mediate part of its pro‐senescence activity through the repression of mitotic genes (Figures 4c,d, Table S6 and S7, and S2). Consistently, we found that this repression occurred as early as 8 hr after inducing depolarization (Figure 4e), whereas other senescence hallmarks were only detectable 2 days after treatment initiation (Figure S8). These data thus support the view that SCN9A‐induced plasma membrane depolarization contributes to OIS by repressing mitotic gene expression.

### 2.6 Plasma membrane depolarization promotes mitotic gene repression through the Rb/E2F pathway

**Figure 5:**
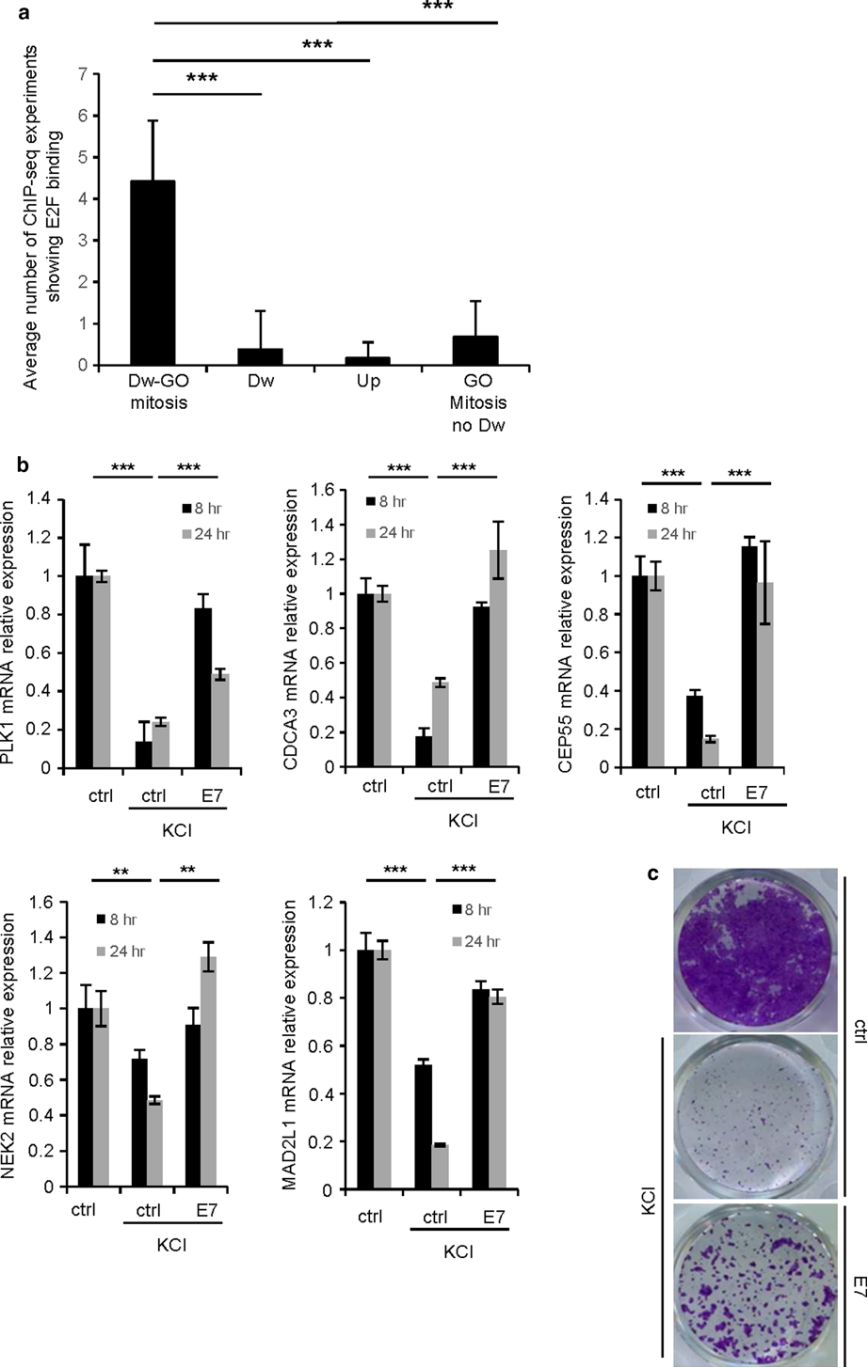
The Rb/E2F pathway mediates plasma membrane depolarization‐induced senescence. (a) The transcription Factor ChIP‐seq Uniform Peaks database from the Encode consortium was explored for seven ChIP‐seq experiments performed on E2F1 or E2F4 transcription factors. We counted the number of experiments showing a ChIP‐seq peak with a minimal score of 500 in the promoter of the top 30 genes identified in transcriptome analysis in four different classes: downregulated mitotic genes (Dw‐GO mitosis); downregulated genes without GO enrichment (Dw); upregulated genes (Up); no downregulated mitotic genes (GO mitosis no Dw). (b,c) HEC‐TM cells were infected with a control (ctrl) or E7 encoding retroviral vector (E7) and selected using neomycin. (b) Cells were then treated or not with 65 mM KCl (KCL) for 8 or 24 hr. RNAs were prepared, RT‐qPCR was performed on the indicated mitotic genes, and the results were normalized against GAPDH mRNA levels. (c) The day after seeding, cells were treated daily with 65 mM KCl. After 8 d, cells were fixed and stained using crystal violet. Experiments were performed at least twice. Statistical analysis was performed with the Student's *t* test, ** means *p* < .01, ***p < .001

Rb and E2F transcription factors are master regulators of cellular senescence. In order to verify whether this pathway could be involved in plasma membrane depolarization‐induced mitotic gene repression, we explored the Encode (The Encyclopedia of DNA Elements) ChIP‐seq database for enrichment of E2Fs binding sites in the promoter regions of genes differentially expressed in response to 24‐hr KCl treatment. Enrichment in E2Fs transcription factor binding sites was only observed in repressed mitotic genes when compared to downregulated nonmitotic genes, upregulated genes, or nonrepressed mitotic genes (Figures 5a and Table S9 and S3) strongly supporting a role for the Rb/E2F pathway in mediating plasma membrane depolarization‐induced mitotic gene repression. To functionally confirm the involvement of this pathway, we prevented the activity of Rb by expressing the E7 protein, which binds to Rb thus restoring the transcription activity of E2F transcription factors (Boyer, Wazer & Band, 1996; Morris et al., 1993). The inhibition of Rb by E7 prevented the repression of mitotic genes induced by plasma membrane depolarization (Figure 5b) and blocked plasma membrane depolarization‐induced senescence (Figure 5c). Taken together, these data demonstrate that plasma membrane depolarization inhibits mitotic gene expression and favors cellular senescence, through the activation of the Rb pathway.

### 2.7 Increased calcium links plasma membrane depolarization to mitotic gene repression

**Figure 6:**
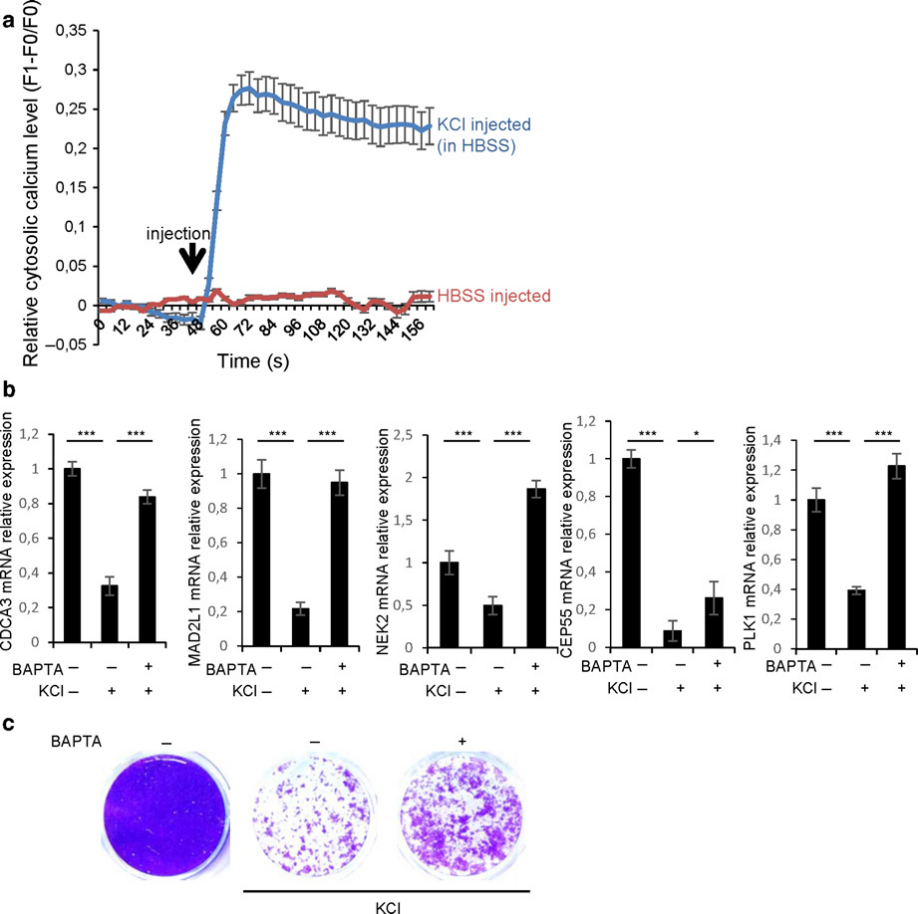
Plasma membrane depolarization favors senescence through increased calcium. (a) HEC‐T cells were incubated with Fluoforte probe to measure cytosolic calcium level with or without KCl injection (65 mM final). The curves represent the mean of three different wells with around 20 cells by wells. (b) Cells were incubated with 2 μM BAPTA‐AM 2 hr before adding KCl (65 mM) for 24 hr. RNAs were prepared, RT‐qPCR was performed on the indicated mitotic genes, and the results were normalized against GAPDH mRNA levels. (c) The day after seeding, cells were treated with 100 nM BAPTA‐AM and 2 hr later, 65 mM KCl was added. Cells were treated daily with KCL and BAPTA‐AM. After 8 d, cells were fixed and stained using crystal violet. The experiments shown are representative of at least two repeats. Statistical analysis was performed with the Student's *t* test, * means *p* < .05, ***p < .001

Plasma membrane depolarization has been shown to increase intracellular calcium (Courtney, Lambert & Nicholls, 1990), and increased calcium can promote cell death and senescence (Borodkina et al., 2016; Roderick & Cook, 2008; Wiel et al., 2014; Martin & Bernard, 2017). We also observed increased calcium after plasma membrane depolarization in HEC‐T cells (Figure 6a). Importantly, mitotic gene repression induced by plasma membrane depolarization was abolished by adding a calcium chelator (BAPTA‐AM) (Figure 6b) strongly supporting that plasma membrane depolarization‐induced mitotic gene repression is mediated by a rise in intracellular calcium. In addition, low doses of BAPTA‐AM calcium chelator were able to prevent proliferation arrest (Figure 6c) supporting an involvement of calcium in mediating plasma membrane depolarization‐induced senescence. These findings support the view that a rise of intracellular calcium mediates mitotic gene repression and senescence induced by plasma membrane depolarization.

## 3 DISCUSSION

Using a loss‐of‐function genetic screen, we identified the SCN9A sodium channel, a gene frequently repressed in tumors, as a pro‐senescence gene, as its knockdown enabled OIS escape. Interestingly, SCN9A levels were upregulated during OIS by the NF‐κB transcription factors. Indeed, we demonstrated that inhibition of NF‐κB transcription factors, either by the constitutive expression of a stabilized IKBA, an endogenous NF‐κB inhibitor, or by knocking down RELA, the main NF‐κB member, suppresses oncogenic stress‐induced SNC9A expression.

NF‐κB transcription factors are well‐known regulators of senescence (Acosta et al., 2008; Bernard et al., 2004; Ferrand et al., 2015). According to our data, the inhibition of NF‐κB transcription factors was sufficient to prevent OIS, corroborating previous findings reported in other senescence contexts (Chien et al., 2011), and this escape from OIS mimicked the one observed following SCN9A knockdown. These data support the view that NF‐κB transcription factors regulate senescence through the regulation of SCN9A and plasma membrane depolarization, together with other known pro‐senescence NF‐κB targets (Acosta et al., 2008; Bernard et al., 2004; Chien et al., 2011; Ferrand et al., 2015; Zdanov et al., 2007).

We determined that the increased expression of SCN9A induced by NF‐κB transcription factors during OIS resulted in plasma membrane depolarization. This plasma membrane depolarization was then shown to mediate senescence, as (i) its reversal by knocking down SCN9A allows to bypass OIS and (ii) forced plasma membrane depolarization induces a premature senescence. It appears that plasma membrane depolarization and SCN9A have so far never been linked to cellular senescence, although it is well established that they impact cell behavior (Chifflet & Hernandez, 2012; Dib‐Hajj, Yang Black & Waxman, 2013; Franco, Bortner & Cidlowski, 2006; Suzuki‐Karasaki, Suzuki‐Karasaki, Uchida & Ochiai, 2014; Willenborg et al., 2011).

Plasma membrane potential depends on the equilibrium between ions both sides of the plasma membrane, and numerous channels are involved in the regulation of this equilibrium. Our results suggest that SNC9A is a key element in regulating OIS, but other factors may also contribute to plasma membrane depolarization and OIS. For example, our microarray analyses by GSEA display enrichment in induction of calcium and potassium voltage‐dependent channels (data not shown) in OIS cells. In addition, an involvement of KCNA1 potassium channel has also been shown to regulate senescence (Lallet‐Daher et al., 2013; Wiel et al., 2014, 2016). Thus, several channels should contribute to plasma membrane potential during OIS, together with SCN9A sodium channels. As during OIS, we also observed that during senescence induced by p53 activation (nutlin treatment), SCN9A expression and plasma membrane depolarization are induced. All these processes are prevented by the knockdown of SCN9A (Figure S10). This suggests that the described SCN9A/plasma membrane potential cascade could be a general mechanism contributing to senescence implementation.

To decipher how plasma membrane depolarization might regulate senescence, we identified the repression of mitotic genes as a bona fide candidate pathway. Indeed, mitotic gene repression was detectable as early as 8 hr after plasma membrane depolarization, long before the appearance of any other senescence hallmarks. Although this repression was detectable in OIS depolarized cells, it was absent in SCN9A knockdown cells upon exposure to an oncogenic stress. Furthermore, almost all of the pathways repressed during forced plasma membrane depolarization were also repressed in OIS cells according to GSEA, reinforcing a major role for plasma membrane depolarization in senescence.

Inhibition of E2F transcription factors by Rb is an important pro‐senescence process (Mallette, Goumard, Gaumont‐Leclerc, Moiseeva & Ferbeyre, 2004; Narita et al., 2003; Sebastian, Malik, Thomas, Sage & Johnson, 2005). Our data support the view that plasma membrane depolarization promotes senescence through the Rb pathway, as repressed mitotic genes are known E2F targets and the inhibition of Rb prevents senescence induced by plasma membrane depolarization. Our data support that Rb/E2F pathway could be activated by a rise of calcium. Importantly, they are emerging evidences for an involvement of increased calcium in senescence. Even if little is known, it has been demonstrated that increased calcium can lead to ROS production and senescence or to calcineurin/NFAT pathway activation and senescence (Wiel et al., 2014; Wu et al., 2010).

In conclusion, our results delineate a new pathway controlled by NF‐κB‐induced SCN9A expression and plasma membrane depolarization, resulting in the activation of the Rb/E2F pathway. These results offer new possibilities for understanding senescence‐associated processes and for developing new tools to manipulate OIS, senescence, and its age‐related disorders.

## 4 MATERIALS AND METHODS

### 4.1 Cell culture and treatment

Human mammary epithelial cells (HECs; Lonza) were cultured in mammary epithelial cell growth medium (Promocell) with penicillin/streptomycin 100 U/ml (Life Technologies). Virus‐packaging GP293 cells (Clontech) were cultured in Dulbecco's modified Eagle medium (DMEM; Life Technologies) supplemented with 10% fetal bovine serum (Life Technologies) and penicillin/streptomycin 100 U/ml. The cells were maintained in a humidified atmosphere at 37°C under 5% CO_2._ HECs were treated daily for 4 days with 4‐OHT (Sigma‐Aldrich) at 250 nM, or with 65 mM KCl (Sigma‐Aldrich), or every 2 days with nutlin‐3 at 1 μM (Sigma‐Aldrich).

### 4.2 Transfection, infection, and vectors

The plasmid pLNCΔMEK1 (ΔN3, S218E, S222D):ER was used to transfer the MEK oncogene. pBabe‐Puro‐IKBalpha‐mut (mIKBA, super‐repressor) was a gift from William Hahn (Addgene plasmid # 15291). Knockdown of SCN9A was performed using Mission Lentiviral Transduction particles targeting SNC9A (TRCN0000424746, TRCN0000426857, TRCN0000433360) green fluorescent protein as control (SHC005V) were purchased from Sigma‐Aldrich. E7‐encoding vector and RELA shRNA were previously described (Acosta et al., 2008). SCN9A overexpression was performed with pCDNA3/SCN9A (a gift of John Wood, UCL, London) and empty pCDNA3 as control. PEI reagent (Euromedex) was used according to the manufacturer's recommendations to transfect GP293 cells with the indicated vector. Two days after transfection, the virus‐containing supernatant was mixed with fresh medium (1/2) and hexadimethrine bromide at 8 μg/ml (Sigma) and used to infect target cells. Infected HECs were selected using G418 (Life Technologies) at 100 μg/ml and/or puromycin (Invivogen) at 500 ng/ml.

### 4.3 Colony formation assay and analysis of senescence‐associated β‐Galactosidase activity

Cells were seeded onto six‐well plates. The following day, several treatments were initiated. The analysis began 6–10 days after the initial treatment. For the colony formation assay, cells were washed with PBS, fixed for 15 min in 3% formaldehyde, and then stained with crystal violet solution. To measure the SA‐β‐Gal activity, cells were washed twice with PBS and fixed for 5 min in 2% formaldehyde/0.2% glutaraldehyde. The cells were then rinsed twice in PBS and incubated at 37°C overnight in SA‐β‐Gal solutions (Augert et al., 2009).

### 4.4 Immunofluorescence

Cells were fixed with 4% paraformaldehyde (PFA) and permeabilized with 0.2% Triton X‐100. The primary antibody against SCN9A (1/200; ASC‐008; Alomone Labs) was incubated at 4°C in TBS + 20% FBS with cells. After three washes, the slides and cells were treated with the anti‐rabbit IgG coupled with Alexa Fluor 488‐labeled (dilution 1/500) diluted in TBS‐FBS. The slides and cells were then washed twice in PBS and counterstained with PBS + Hoechst (1/1,000) for 10 min at room temperature. The slides were then mounted with Fluoromount‐G (Cliniscience).

### 4.5 Reverse transcription and real‐time quantitative PCR

TRI Reagent (Sigma‐Aldrich) was used to perform phenol–chloroform extractions of total RNA. The First‐Strand cDNA Synthesis Kit (GE Healthcare, Chalfont St Giles, UK) was used to synthesize cDNA from 2 μg of total RNA, according to the manufacturer's instructions. The RT reaction mixture was diluted 1/20 and used as a cDNA template for the qPCR. A TaqMan quantitative PCR was carried out on a FX96 Thermocycler (Bio‐Rad). The PCR mixture contained TaqMan mix (Roche), 200 nM of primers, the Universal Probe Library probe (100 μM) for the gene of interest (TaqMan Gene Expression Assays (Primers/probe), Life technologies) added up with 1.67 μl of cDNA template. All of the reactions were performed in triplicate. The relative amount of mRNA was calculated using the comparative Ct (ΔΔCT) method, following data normalization against 1 housekeeping gene. The PCR primers used for the qPCR are available upon request.

### 4.6 Plasma membrane potential measurement

Variations in the plasma membrane potential were measured according to two distinct approaches. In the first, cells were washed three times with PBS 1× and the cell pellet was suspended in PBS 1× containing the 200 nM of the anionic membrane voltage‐reporting dye DiBaC4 (Molecular Probes, stock solution at 10 mg/ml in dimethyl sulfoxide). The cells were loaded by incubation with 1 ml DiBAC4 dye at 37°C for 1 hr. The fluorescence intensity was measured with a FACSCalibur flow cytometer (BD Bioscience) at 525 nm, after excitation at 488 nm, and the fluorescence data were analyzed with the FlowJo software.

Alternatively, plasma membrane depolarization was evaluated by electrophysiological recordings carried out at room temperature (20–23°C) in the conventional whole‐cell configuration of the patch‐clamp technique (Hamill, Marty, Neher, Sakmann & Sigworth, 1981). The internal solution contained (in mM): 130 KCl, 1 MgCl2, 3 MgATP, 5 HEPES, adjusted to pH 7.2 with KOH and the external solution contained (in mM): 140 NaCl, 4 KCl, 2 MgCl_2_, 2.5 CaCl2, 10 glucose, 10 HEPES, adjusted to pH 7.4 with NaOH. Membrane currents were evoked from a holding potential of −80 mV, by voltage ramps of 1.5‐s duration, applied from −100 to +90 mV, sampled at 1 kHz, and filtered at 300 Hz. Reversal potentials of ramp membrane currents were determined using linear fits extrapolated on‐ramp currents in a potential range around the zero current, and it was verified in the I0 current‐clamp mode of the patch‐clamp amplifier that they were close to the membrane potentials of cells.

### 4.7 Transcriptome and bioinformatics analysis

Transcriptome analysis of HEC‐TM cells, treated or not with either 4‐OHT (100 nM) or KCl (65 mM), was performed using Whole Human Genome Oligo 4 × 44 K Microarrays (Agilent Technologies) and the one‐color gene expression Agilent workflow. Briefly, cRNAs were synthesized and labeled with the Cy3 dye from 100 ng of total RNA using the one‐color Low Input Quick Amp Labeling Kit (Agilent Technologies). Then, 1,650 ng of Cy3‐labeled cRNAs purified using the RNeasy Mini‐spin columns (Qiagen) was hybridized on the 4 × 44 K arrays for 17 hr at 65°C. Microarrays were washed and scanned with an Agilent DNA microarray scanner G2565CA (Agilent Technologies). Fluorescent signals were extracted and normalized with the Feature Extraction software version 10.5.1.1 (Agilent Technologies), and transferred to the GeneSpring GX 12.6 software (Agilent Technologies) for data processing and data mining. All of the conditions were tested in three independent biological replicates (*n* = 3) for statistical analyses. Microarray probes were filtered using the Agilent flag filter to remove probes with a raw signal below 30 in all of the conditions tested. Genes differentially expressed between treated HEC‐TM cells, and untreated cells were defined using unpaired *t* test *p* value <.01 with a Benjamini–Hochberg correction and fold change cutoffs > or <2, for up‐ and downregulation, respectively.

For data visualization, hierarchical clustering was performed using the Euclidian metric and complete linkage method.

The GO tool from *GeneSpring* enabled us to determine statistically significant enrichments in biological processes, based on computation *p*‐values described by standard hypergeometric distribution.

A Gene set enrichment analysis (GSEA) was performed from gene expression data generated in each experimental condition, using the “preranked” tool (Subramanian et al., 2005). GSEA determines whether GO set of genes shows statistically significant differences between control and KCl‐24 hr or 4‐OHT‐96 hr conditions. The input data for GSEA procedure were the following: (i) a complete table of genes ranked according to the log2‐transformed fold change expression, (ii) a mapping file for identifying transcripts in the corresponding platform, and (iii) 1,039 functional gene sets corresponding to GO pathways available from the Molecular Signature Database. Default parameters were used. Inclusion gene set size was set between 5 and 500, and the phenotype was permutated 1,000 times.

An enrichment score was also computed in unique samples for a given gene set, using the ssGSEA tool (Barbie et al., 2009). We ran ssGSEA from GenePattern (Reich et al., 2006) to compute the enrichment score of a given pathway in each sample. Using this tool, the gene expression values for a given sample were rank‐normalized, and an enrichment score was produced using the empirical cumulative distribution functions (ECDF) of the genes in the gene set and the remaining genes. Before running ssGSEA, we used the “Collapse Dataset” module to collapse all probe set expression values for a given gene into a single expression value, using the median.

### 4.8 Live calcium imaging

HEC‐T cells cultured in NUC LabTek chambered cover glass were incubated with 2 μM Fluoforte probe (Enzo Life Science) during 30 min in Hank's balanced salt solution with 2 mM calcium to study cytosolic calcium level. Fluorescence values were measured using Zeiss LSM 780 confocal microscope. For the measurement, cells were incubated in calcium‐free HBSS medium. After few seconds, KCl in calcium‐free HBSS was injected to have 65 mM final. For the control, HBSS was injected without KCl.

### 4.9 Statistical analysis

In graphs, values are mean ± *SD* (standard deviation) for three independent experiments. * means *p* < .05; **p < .01; ***p < .001 correspond to *p*‐value according to the Student's *t* test.

## CONFLICT OF INTEREST

None declared.

## AUTHOR CONTRIBUTIONS

MW, JMF, NM, and DB designed research; MW, JMF, CC, DV, CW, BG, EB, PM, FVC, AG and PS performed research; MW, JMF, CC, CW, BG, DV, and JPF analyzed data; MW, JMF, and DB wrote the article; and DB supervised the all research.
