# Supplementary material for: The SCN9A channel and plasma membrane depolarization promote cellular senescence through Rb pathway
Source: Aging Cell. 2018 Feb 15;17(3):e12736. doi: 10.1111/acel.12736 (PMC5946064; doi:10.1111/acel.12736)
Supplement: Supplementary file 1 [file ACEL-17-e12736-s001.pdf]

Supplementary Figure Legends

Figure 1 Identified regulators of senescence. (a-c) Normal human diploid fibroblasts were infected with a PDGFB encoding retroviral vector and shRNA library pools (10,000 shRNAs per pool and in 7 pools, DecodeTM RNAi viral screening pools, Thermo Scientific) and selected. shRNA sequences of the clones escaping PDGFB-induced senescence were identified as previously described (24, 26). Independent shRNA against each identified gene (POLB, YWHAG, CTSA, CASP3, LONP2, JAM3, SCN9A and PRKDC) was used to infect together with PDGFB normal human diploid fibroblasts. (a) Knockdown effect of these shRNA were verified by RT-qPCR on the infected cells. (b) SA-β-Gal assays were performed to demonstrate the impact of shRNA against identified genes on senescence in PDGFB-expressing cells. (c) Description of senescence regulators identified in this study.

Figure 2 Validation of SCN9A knockdown. (a) U2OS were transfected by either empty pCDNA3 or pCDNA3/SCN9A (gift of John Wood, UCL, London). Two days after, cells were fixed and immunofluorescence staining using anti-SCN9A antibody was performed. Nuclei were counterstained using Hoechst dye. (b) HEC-TM cells were infected with a control shRNA (ctrl) or with three independent shRNA encoding retroviral vectors against SCN9A (shRNA_SCN9A A, B, C). Immunofluorescence against SCN9A was performed. Experiments were performed at least 2 times.

Figure 3 NF-κB mediates increased SCN9A expression and OIS. (a) HEC-TM cells were infected with a shRNA control (ctrl) or two distinct shRNA encoding retroviral vector against NF-κB subunit, RELA (shRNA_RELA 1, 2). After RNA extraction, RNAs were prepared and

RELA was measured by RT-qPCR, using GAPDH as reference. (b-g) One day after seeding, cells were treated daily with 4-OHT for 4 days. (b) RNAs were prepared and SCN9A level was examined by RT-qPCR using GAPDH as reference. (c) Immunofluorescences against SCN9A were performed. Representative images are shown. (d) Five days later, cells were fixed and stained with crystal violet for colony assays. (e) Cells were fixed and SA-β-Gal staining performed. The histogram displays the percentage of positive cells in the indicated experimental conditions. (f-g) RNAs were prepared and RNA levels of (f) IL8 or (g) IL6 were measured by RT-qPCR in triplicate and normalized to GAPDH levels. Experiments in this figure are representative of at least 2 independent experiments. Statistical analysis was performed with the student's *t-test*, *** means $p<0.001$.

Figure 4 NF-κB inhibition reduces plasma membrane depolarization during OIS. (a) HEC-TM cells were infected with a control vector (ctrl) or with mIKBA cDNA encoding retroviral vectors (mIKBA) and selected. Flow cytometry profiles of cells treated daily during 4 days by 4-OHT and stained with DiBAC4 probe. (b) Histogram showing mean of DiBAC4 fluorescence values from cytometry analysis in triplicate. Statistical analysis was performed with the student's *t-test*, *** means $p<0.001$. Two fully independent experiments have been carried out.

Figure 5 KCl-induced senescence is not impacted by loss of SCN9A. One day after seeding, HEC-TM cells expressing or not shRNA against SCN9A were treated by 4-OHT, KCl or both. Six days later, cells were fixed and crystal violet stained. This experiment is representative of 2 independent experiments.

Figure 6 Heat Map representing microarray data for 78 GO M_Phase genes repressed commonly in KCl-24h and 4-OHT-96h conditions. Pseudocolors indicate differential expression of GO M_Phase genes in three different conditions; (4-OHT-96h) HEC-TM treated four days with 4-OHT; (KCl-24h) HEC-TM treated one day with KCl; (4-OHT-96h shSCN9A) HEC-TM cells infected with shRNA against SCN9A treated four days with 4-OHT. Data were normalized to HEC-TM untreated cells condition (ctrl) (Green indicates down-regulated genes and red up-regulated transcripts).

Figure 7 Validation of mitotic gene repression during OIS and the reversal following SCN9A knockdown. HEC-TM cells were infected with a shRNA control (ctrl) or with three distinct shRNA against SCN9A encoding retroviral vectors (shRNA_SCN9A A, B, C). After 4 days of 4-OHT treatment, RNA was purified and RT-qPCR was performed for the indicated mitotic genes: (a) *CDCA3*, (b) *MAD2L1*, (c) *NEK2*, (d) *CEP55*, and (E) *PLK1*. Values indicate mean ratio expression of triplicate using *GAPDH* as reference. Experiments were performed 3 times. Statistical analysis was performed with the student's *t-test*, ** means $p<0.01$, *** $p<0.001$.

Figure 8 Kinetics of senescence induced by plasma membrane depolarization. HEC-TM cells were treated daily with 65 mM of KCl. At the indicated times, cells were either (a) fixed for SA-β-Gal activity measurement or (b) processed for RNA extraction for analysis of IL6 mRNA levels by RT-qPCR using GAPDH as reference. Experiments were performed twice Statistical analysis of triplicate was performed with the student's *t-test*, ** means $p<0.01$, *** $p<0.001$.

Figure 9 Analysis of E2F transcription factor binding sites using the Encode database. UCSC browser screenshot of the promoter regions of genes of interest, showing ChIP-seq analysis of binding sites for E2F1 and E2F4 transcription factors according to the Encode database using a score >500. UCSC genes and genomic coordinates referring to hg19 assembly are shown. One gene of interest per class of examined gene is shown: down-regulated mitotic genes (Dw-GO mitosis); down-regulated genes without GO enrichment (Dw); up-regulated genes (Up); no down-regulated mitotic genes (GO mitosis no Dw).

Figure 10 Loss of SCN9A inhibits nutlin-induced senescence. HEC-TM expressing or not a shRNA directed against SCN9A were treated every 2 days with nutlin-3 at 1μM. (a-b) Four days later, RNAs were prepared, reverse transcribed (RT) and the SCN9A (a) or p21 (b) transcripts were quantified by qPCR. Results were normalized with respect to the level of GAPDH transcript. (c) Seven days later, cells were fixed and stained using crystal violet. (d) Four days later, RNAs were prepared, reverse transcribed, and Ki67 transcripts were quantified by quantitative PCR. (e) Cells were stained for SA-β-Gal activity and the number of SA-β-Gal positive cells was counted in each condition. (f) Cells were incubated with the fluorescent DiBAC4 dye to measure their relative plasma membrane potential (ΔV(m)) by flow cytometry. Histograms showing the mean relative DiBAC4 fluorescence are shown. The experiments shown are representative of at least 2 biological repeats. Statistical analysis was performed with the student's *t-test*, * means $P<0.05$, ** $P<0.01$, *** $P<0.001$.

Supplemental Figure 1, Warnier et al

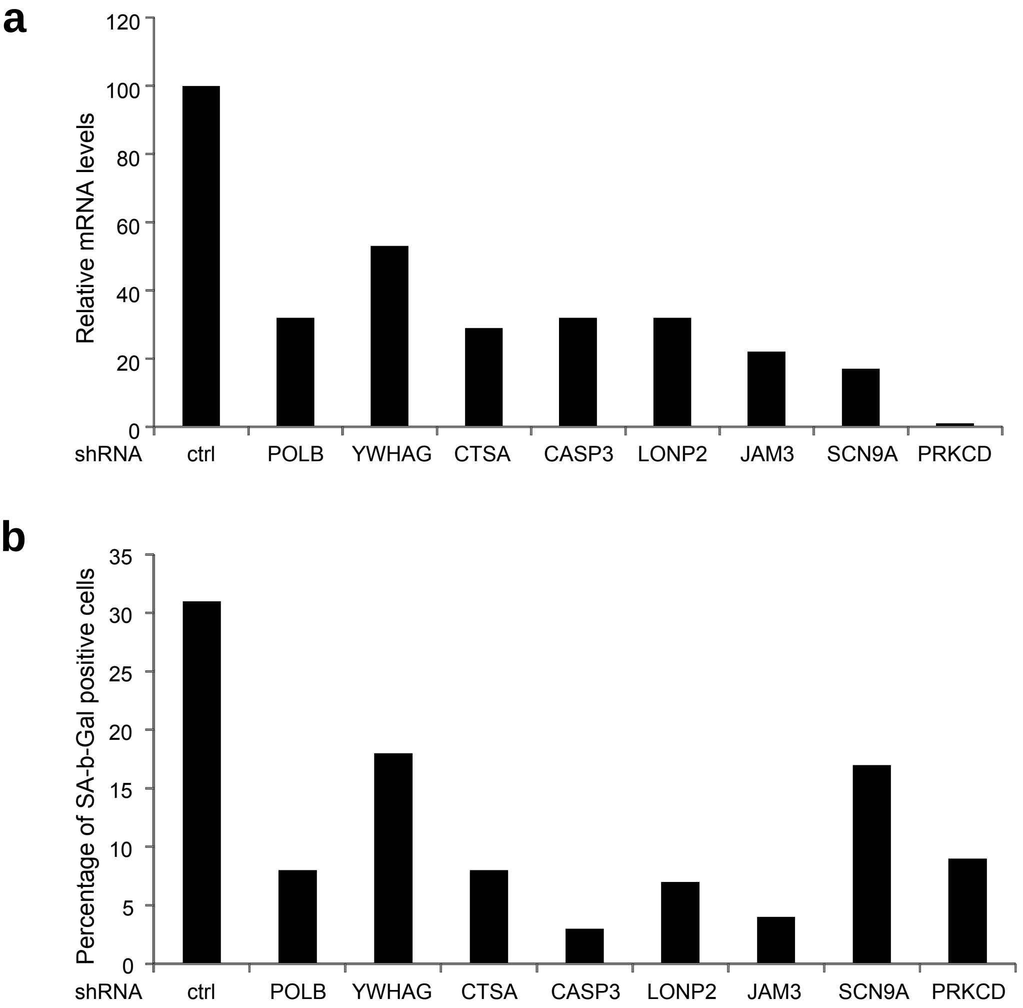

c

List of senescence regulators	
CASP3	Caspase 3
CTSA	Cathepsin A
JAM3	Junctional Adhesion Molecule 3
LONP2	Lon Peptidase 2
POLB	Polymerase (DNA Directed). Beta
PRKDC	Protein Kinase, DNA-Activated, Catalytic Polypeptide
**SCN9A**	**Sodium Channel. Voltage Gated. Type IX Alpha Subunit**
YWHAG	Tyrosine 3-Monooxygenase/Tryptophan 5-Monooxygenase Activation Protein. Gamma

Supplemental Figure 2, Warnier et al

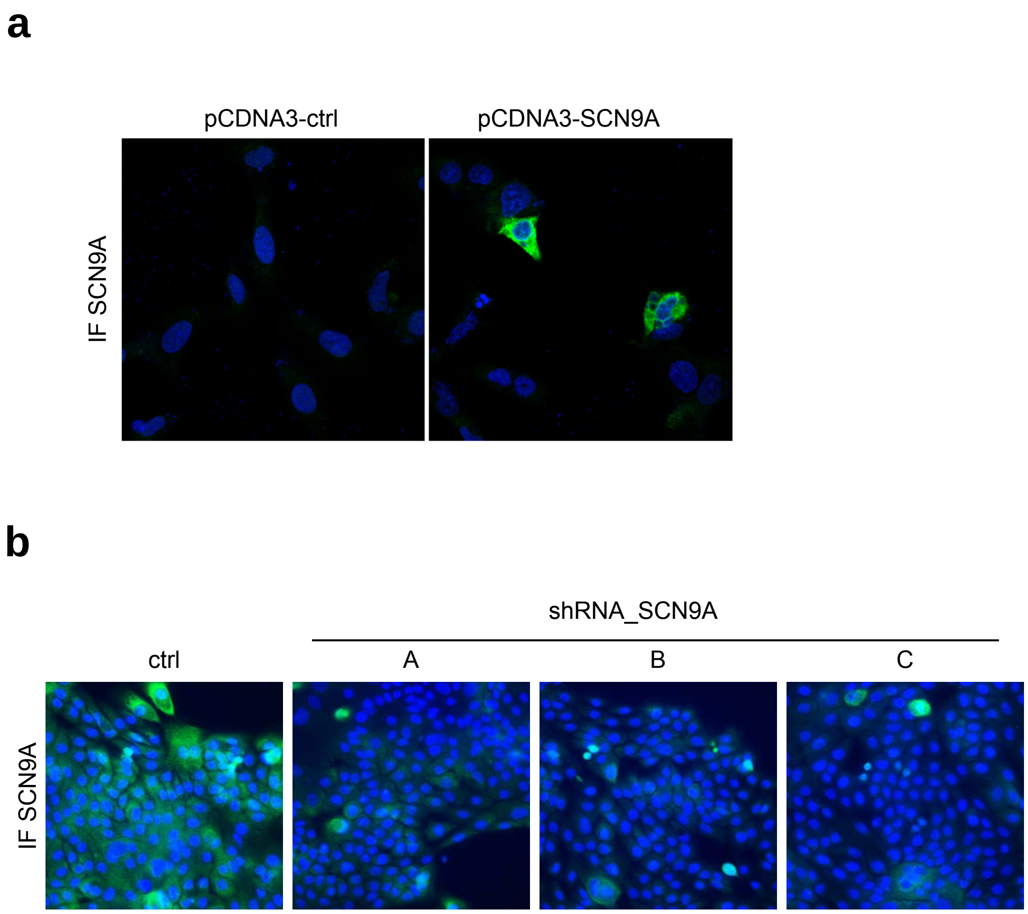

Supplemental Figure 3, Warnier et al

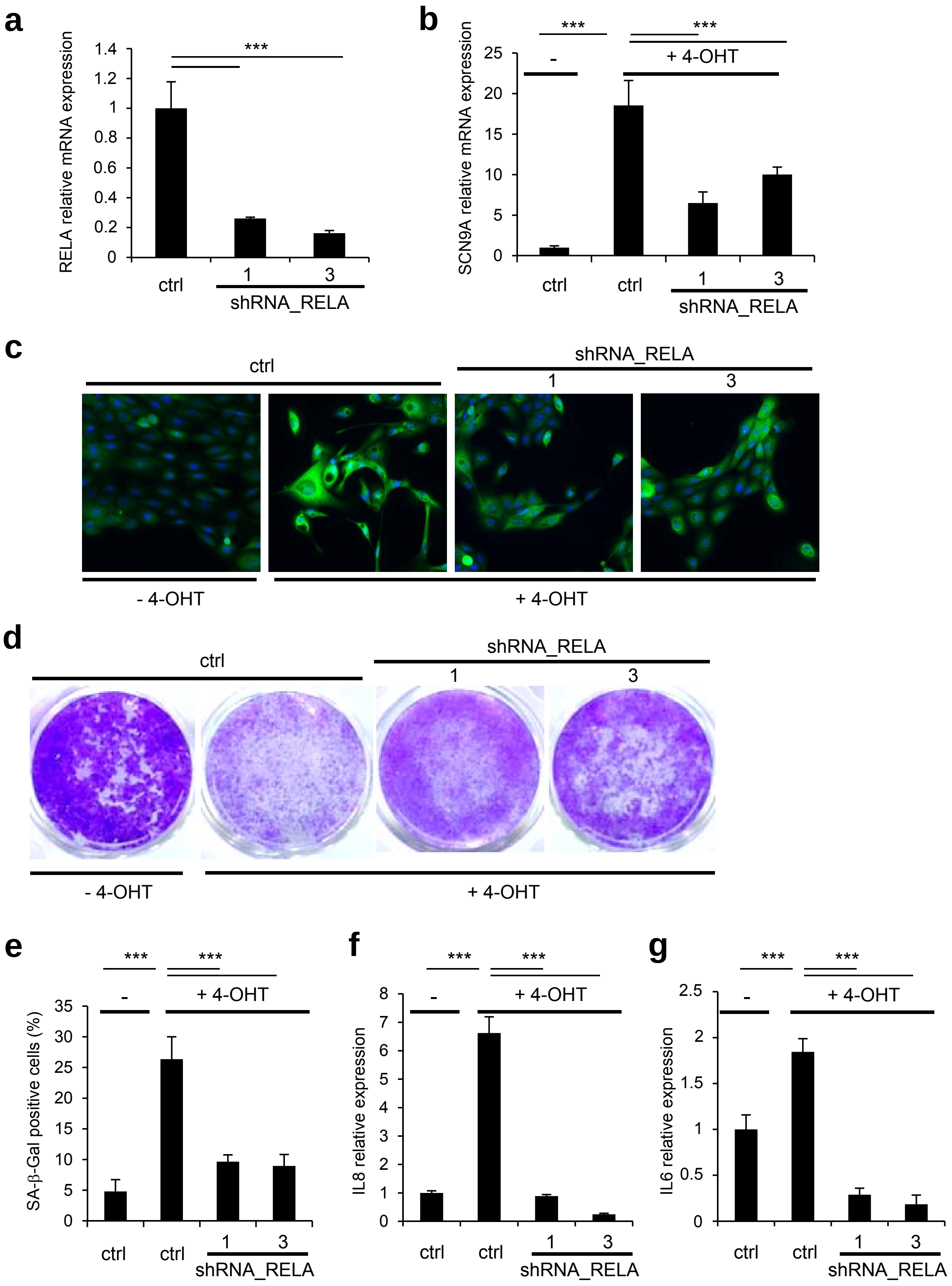

Supplemental Figure 4, Warnier et al

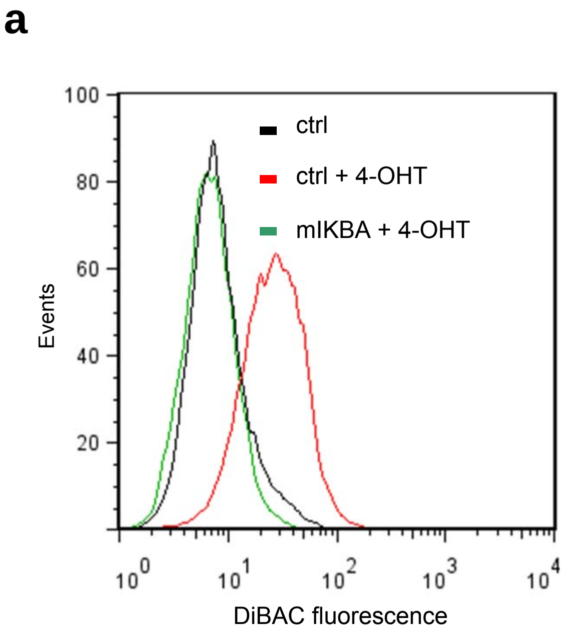

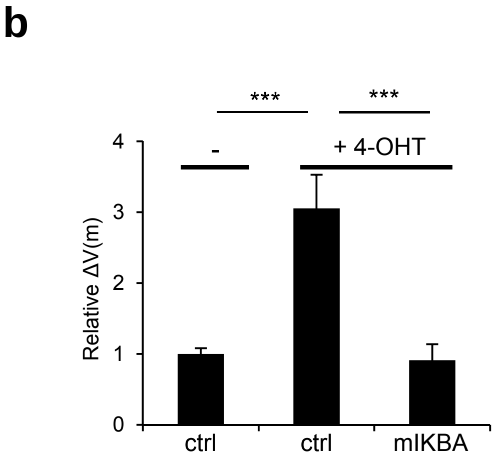

Supplemental Figure 5, Warnier et al

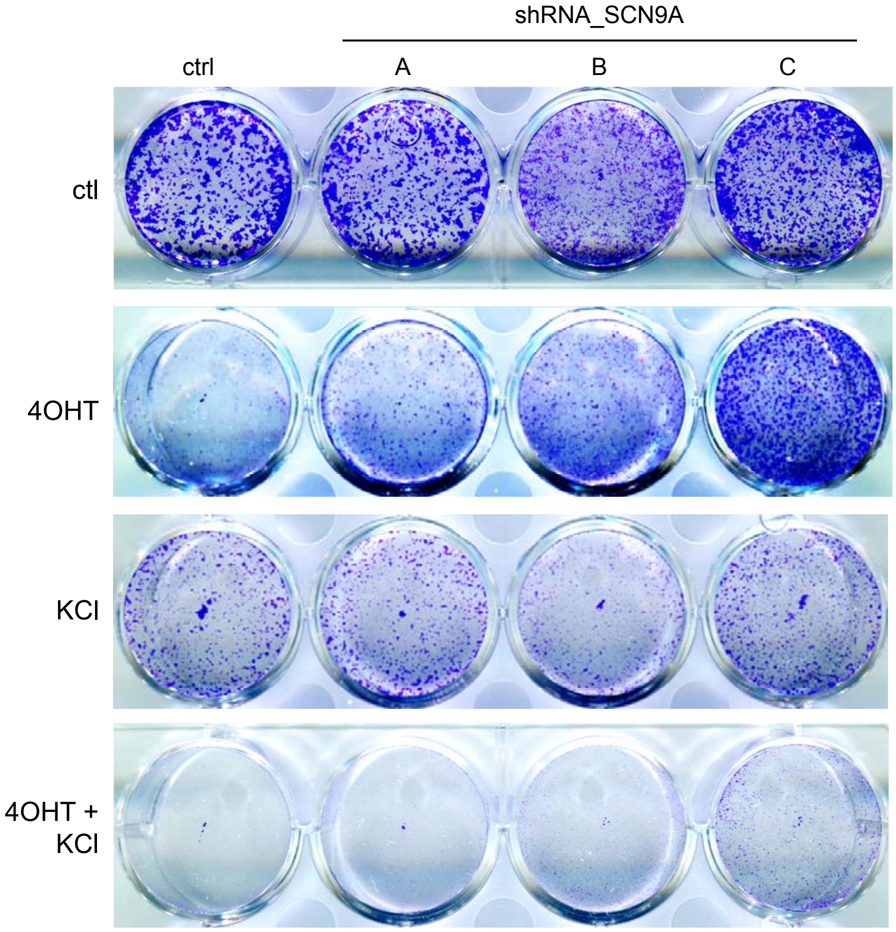

Supplemental Figure 6, Warnier et al

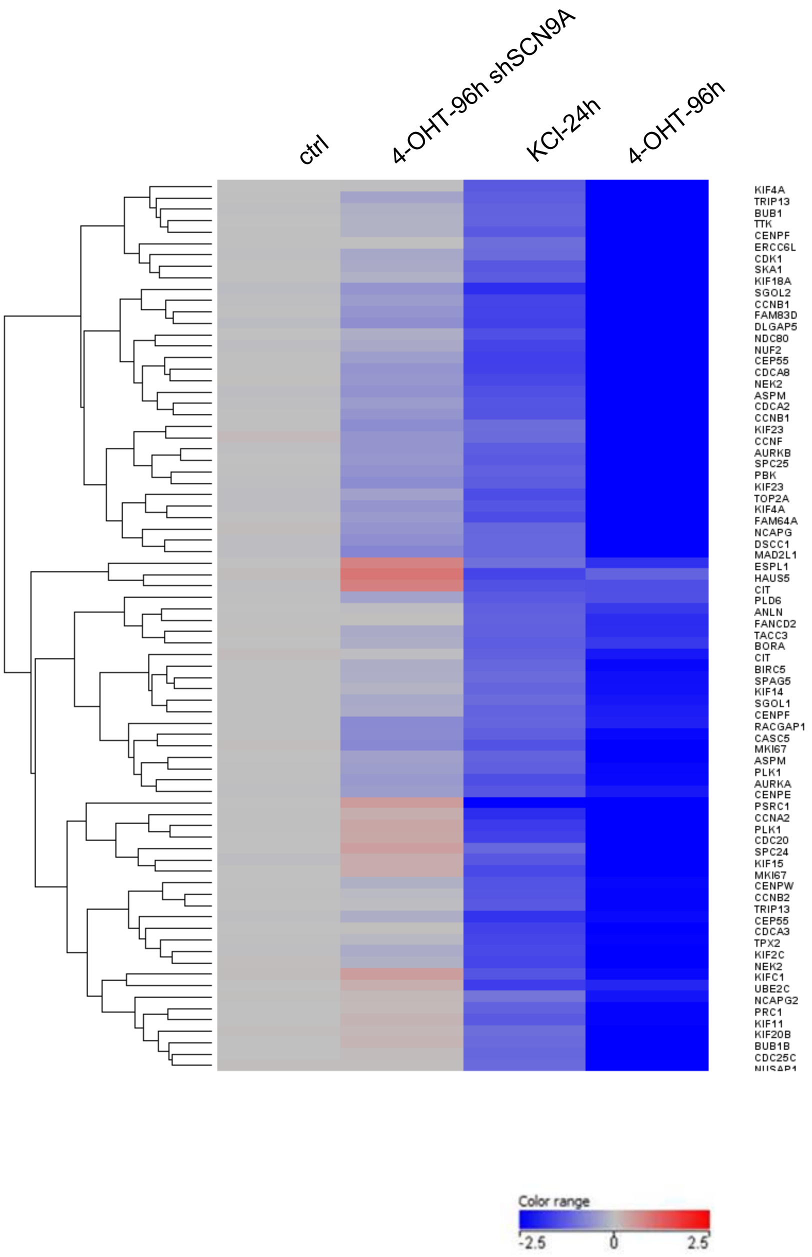

Supplemental Figure 7, Warnier et al

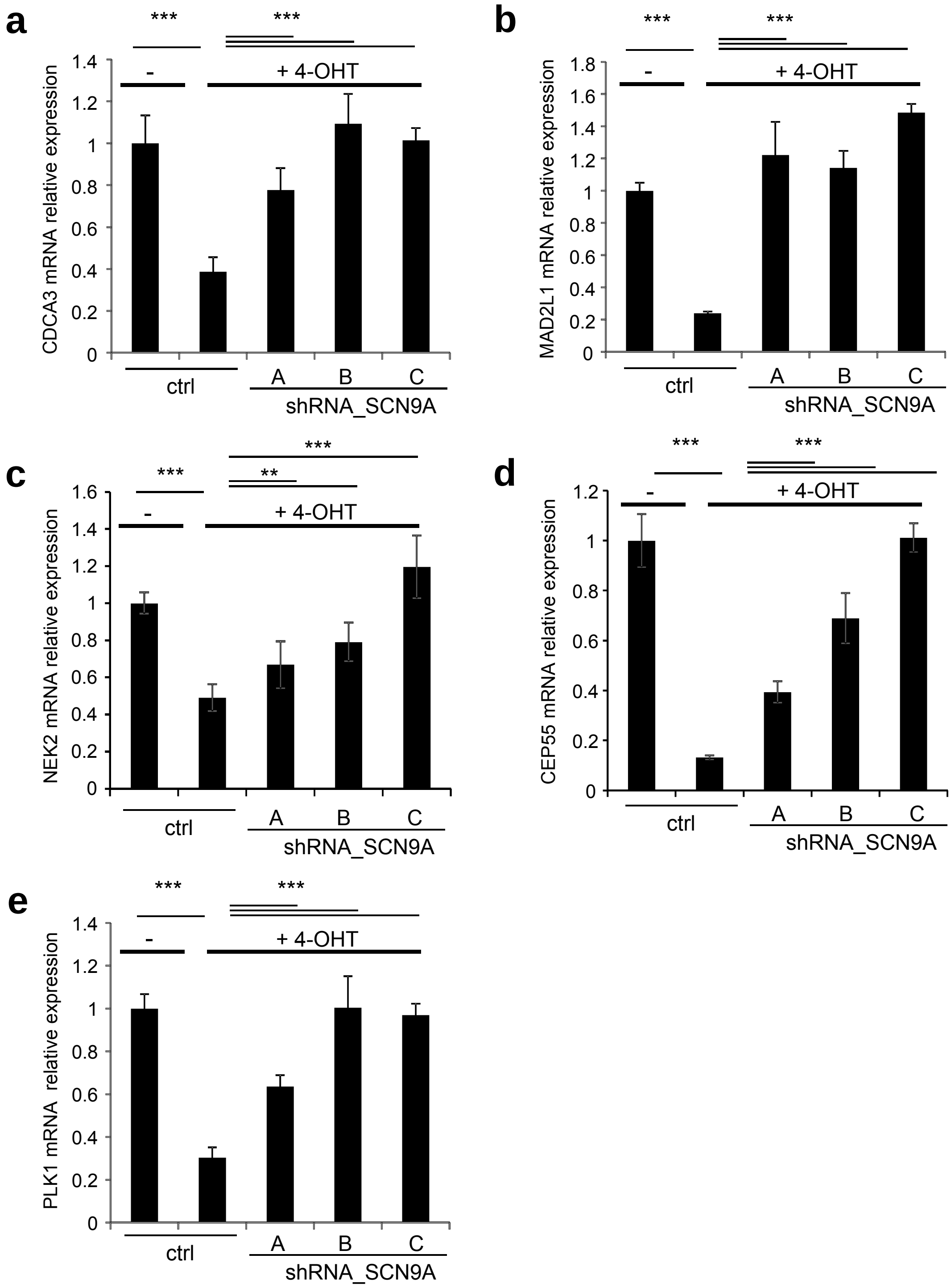

Supplemental Figure 8, Warnier et al

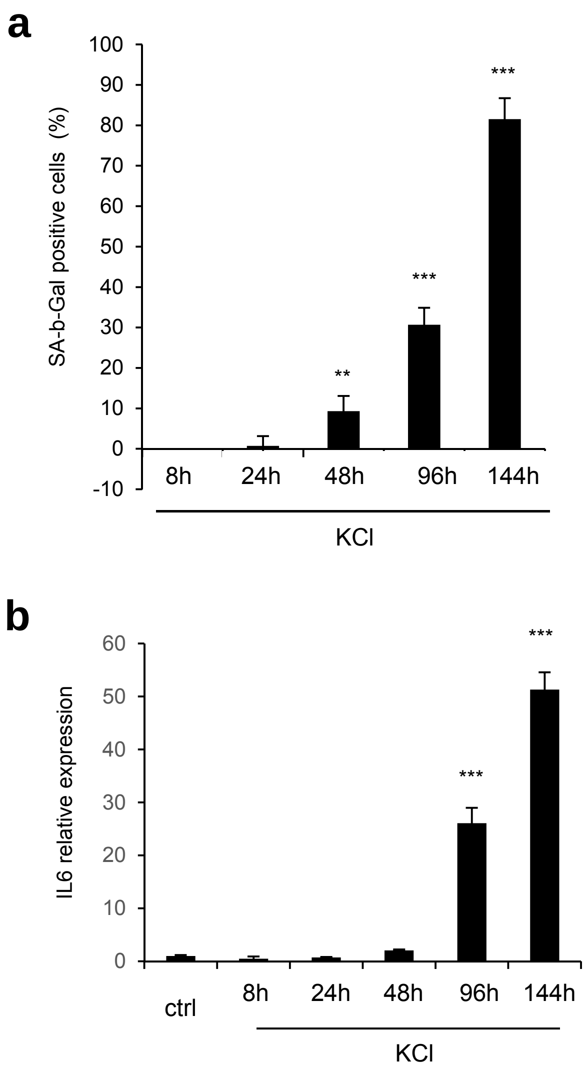

Supplemental Figure 9, Warnier et al

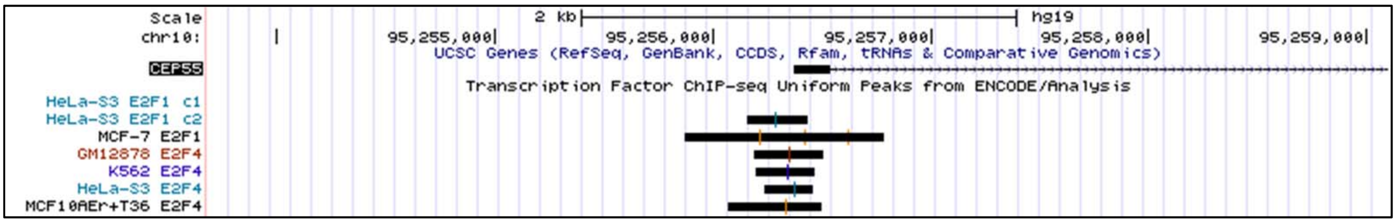

Dw-GO mitosis

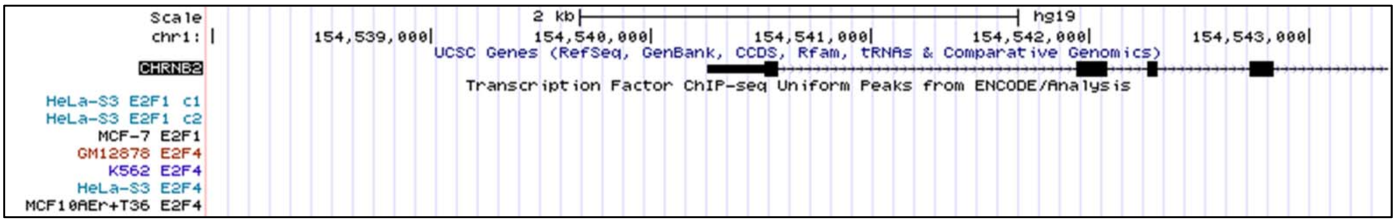

Dw

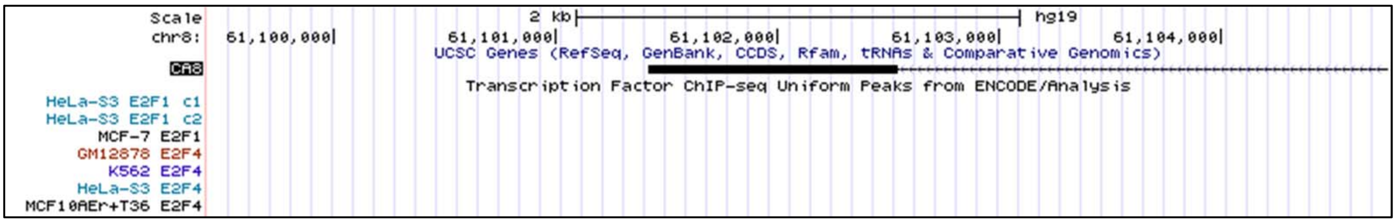

Up

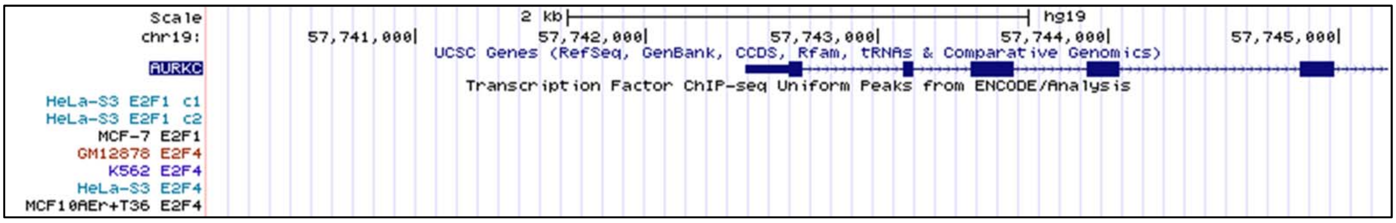

GO mitosis no dw

Supplemental Figure 10, Warnier et al

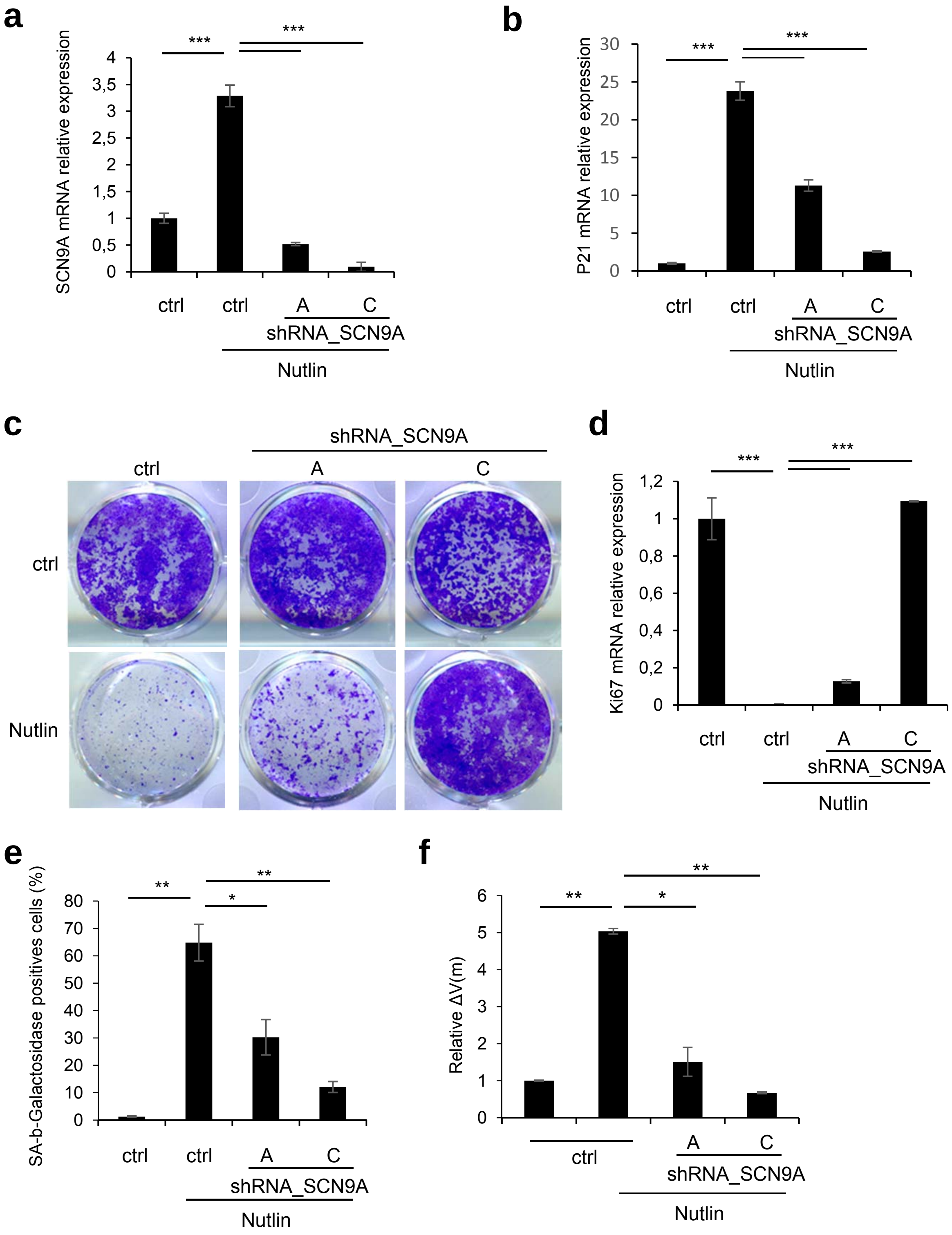

GO ACCESSION	GO Term	Corrected p-value	Count in Selection	% Count in Selection	Count in Total	% Count in Total	Enrichment
GO:0000087	M phase of mitotic cell cycle	8.87E-32	60	14.7	332	1.9	7.9
GO:0007067	mitosis	1.10E-31	59	14.5	326	1.8	7.9
GO:0000280	nuclear division	1.10E-31	59	14.5	326	1.8	7.9
GO:0000279	M phase	1.19E-31	69	16.9	473	2.7	6.3
GO:0048285	organelle fission	6.64E-31	60	14.7	353	2.0	7.4
GO:0000278	mitotic cell cycle	1.21E-27	79	19.4	732	4.1	4.7
GO:0022403	cell cycle phase	4.42E-27	77	18.9	709	4.0	4.7
GO:0022402	cell cycle process	1.56E-24	83	20.3	900	5.1	4.0
GO:0007049	cell cycle	3.57E-24	96	23.5	1205	6.8	3.5
GO:0051301	cell division	4.98E-24	58	14.2	439	2.5	5.7
GO:0007059	chromosome segregation	2.27E-20	36	8.8	177	1.0	8.8
GO:0000779	condensed chromosome, centromeric region	3.36E-17	26	6.4	98	0.6	11.5
GO:0000793	condensed chromosome	1.38E-16	33	8.1	186	1.0	7.7
GO:0005819	spindle	9.62E-16	38	9.3	272	1.5	6.1
GO:0000777	condensed chromosome kinetochore	9.92E-16	24	5.9	91	0.5	11.5
GO:0000775	chromosome, centromeric region	3.43E-14	29	7.1	166	0.9	7.6
GO:0000819	sister chromatid segregation	9.53E-14	21	5.1	78	0.4	11.7
GO:0000776|GO:0005699	kinetochore	2.91E-13	24	5.9	115	0.6	9.1
GO:0000070|GO:0016359	mitotic sister chromatid segregation	5.96E-13	20	4.9	75	0.4	11.6
GO:0051726|GO:0000074	regulation of cell cycle	5.63E-11	57	14.0	802	4.5	3.1
GO:0010564	regulation of cell cycle process	5.97E-11	41	10.0	443	2.5	4.0
GO:0007346	regulation of mitotic cell cycle	5.97E-11	37	9.1	363	2.0	4.4
GO:0051783	regulation of nuclear division	1.24E-10	20	4.9	98	0.6	8.9
GO:0007088	regulation of mitosis	1.24E-10	20	4.9	98	0.6	8.9
GO:0000226	microtubule cytoskeleton organization	1.70E-10	33	8.1	301	1.7	4.8
GO:0000780	condensed nuclear chromosome, centromeric region	4.18E-10	10	2.5	16	0.1	27.2
GO:0007051	spindle organization	4.58E-10	19	4.7	93	0.5	8.9
GO:0005694	chromosome	5.97E-10	54	13.2	780	4.4	3.0
GO:0006996	organelle organization	8.95E-10	115	28.2	2591	14.6	1.9
GO:0015630	microtubule cytoskeleton	1.88E-09	60	14.7	959	5.4	2.7
GO:0007017	microtubule-based process	3.40E-09	39	9.6	462	2.6	3.7
GO:0007052	mitotic spindle organization	5.31E-09	14	3.4	50	0.3	12.2
GO:0005874	microtubule	8.45E-09	34	8.3	370	2.1	4.0
GO:0030496	midbody	3.08E-08	19	4.7	118	0.7	7.0
GO:0044430	cytoskeletal part	3.29E-08	73	17.9	1402	7.9	2.3
GO:0051276|GO:0007001|GC	chromosome organization	4.28E-08	52	12.7	824	4.6	2.7
GO:0044427	chromosomal part	9.87E-08	46	11.3	689	3.9	2.9
GO:0033043	regulation of organelle organization	1.09E-07	44	10.8	642	3.6	3.0
GO:0000922|GO:0030615	spindle pole	1.09E-07	18	4.4	113	0.6	6.9
GO:0008017	microtubule binding	1.19E-07	22	5.4	176	1.0	5.4
GO:0043228	non-membrane-bounded organelle	1.35E-07	136	33.3	3556	20.1	1.7
GO:0043232	intracellular non-membrane-bounded organelle	1.35E-07	136	33.3	3556	20.1	1.7
GO:0070925	organelle assembly	2.45E-07	20	4.9	150	0.8	5.8
GO:0005876	spindle microtubule	3.02E-07	13	3.2	56	0.3	10.1
GO:0051303	establishment of chromosome localization	5.45E-07	12	2.9	48	0.3	10.9
GO:0050000	chromosome localization	5.45E-07	12	2.9	48	0.3	10.9
GO:0071842	cellular component organization at cellular level	5.45E-07	144	35.3	3928	22.2	1.6
GO:0000778	condensed nuclear chromosome kinetochore	8.42E-07	6	1.5	7	0.0	37.2
GO:0031577	spindle checkpoint	8.93E-07	11	2.7	40	0.2	11.9

Table S1. Gene Ontology analysis showing the list of pathways shared by genes downregulated during OIS and plasma membrane depolarization using GeneSpring.

ProbeName	FC ([oht96] vs [shc])	FC ([k24] vs [c])	[c](normalized)	[k24](normalized)	[oht96](normalized)	[shc](normalized)	GeneSymbol
A_23_P10182	-4.3404365	-2.0379615	0.012804031	-1.0143228	-1.3346109	0.78322905	ACOX2
A_23_P210482	-3.243032	-2.0075479	0.003455162	-1.0019792	-1.8776541	-0.18031089	ADA
A_33_P3330503	-7.292356	-2.656408	0.021935144	-1.3875417	-2.5843918	0.28199324	ALDH7A1
A_33_P3330498	-11.784073	-2.4493136	-0.07006407	-1.3624415	-2.7422955	0.8164708	ALDH7A1
A_23_P356684	-3.290487	-2.4088664	-7.966359E-4	-1.2691511	-1.7614483	-0.043147087	ANLN
A_24_P225468	-2.5681546	-2.205996	0.00646464	-1.1349655	-1.2185917	0.14214039	ANP32E
A_23_P77304	-2.2924232	-2.1308625	-0.04926427	-1.1407018	-1.3254071	-0.12853368	AP3B2
A_24_P296254	-7.983251	-3.0725324	-0.002960682	-1.622389	-3.5645268	-0.56755036	ARHGAP11A
A_33_P3339361	-6.4064713	-3.6613815	0.015187581	-1.8572005	-2.717801	-0.03827111	ARHGAP11A
A_33_P3339375	-3.5427008	-2.3284032	0.017372767	-1.2019682	-1.959179	-0.13432948	ARHGAP11B
A_23_P404730	-3.019224	-2.0577242	-0.018398127	-1.0594478	-0.47903633	1.1151414	ARHGAP33
A_24_P274615	-2.4353726	-3.1675456	-0.0015800794	-1.6649455	-1.0867453	0.19739723	ARRDC3
A_33_P3288159	-4.7385683	-2.6740787	-0.039662044	-1.458704	-2.8439255	-0.59947425	ASPM
A_23_P52017	-4.3317285	-2.315265	0.0059986115	-1.2051786	-2.5254376	-0.4104948	ASPM
A_23_P131866	-3.6934907	-2.7596807	-0.014659882	-1.4791613	-2.3925664	-0.5075814	AURKA
A_23_P130182	-5.331205	-2.4122612	-0.024249077	-1.2946352	-2.9797242	-0.5652625	AURKB
A_23_P154086	-3.5872018	-2.0801673	3.4650166E-5	-1.0566648	-1.6890932	0.15376568	BCS1L
A_23_P118815	-4.4931307	-2.2583432	0.0069704056	-1.1682943	-2.417779	-0.25005785	BIRC5
A_23_P25626	-2.8787642	-2.4451127	-0.0016902288	-1.2915913	-1.7720355	-0.24658585	BORA
A_23_P124417	-5.409993	-2.2877998	-0.024033865	-1.2179947	-2.6398487	-0.20422204	BUB1
A_23_P163481	-6.419632	-2.161234	-0.00206693	-1.1139221	-2.5228772	0.1596133	BUB1B
A_23_P141520	-2.3611336	-2.418583	-0.0030733745	-1.2772354	-0.9607792	0.2787005	C17orf49
A_23_P32135	-2.3377807	-2.764045	0.025808811	-1.4409723	-0.80005294	0.42508665	C9orf9
A_23_P4944	-2.6081464	-2.0170143	0.0051695504	-1.0070518	-0.554965	0.82805985	CALM3
A_23_P100127	-3.2869856	-2.4222727	0.007440885	-1.2689203	-2.4125261	-0.695761	CASC5
A_23_P40453	-3.484541	-2.8294942	0.003353119	-1.4971911	-1.972123	-0.17115434	CBR3
A_24_P157156	-3.871548	-2.3082662	-0.006433487	-1.213243	-2.2420676	-0.28915706	CCDC150
A_33_P3327165	-2.5996668	-2.2498627	0.023102442	-1.1467346	-1.3265505	0.05177625	CCDC18
A_23_P58321	-8.83199	-3.789821	0.00789388	-1.9142357	-2.8997014	0.24303722	CCNA2
A_23_P122197	-5.5635943	-2.9916422	-0.029526075	-1.6104637	-2.9584217	-0.48240438	CCNB1
A_33_P3401621	-4.478595	-2.6751537	0.014121373	-1.4055004	-2.7178621	-0.55481595	CCNB1
A_23_P65757	-5.202039	-2.6538603	0.017891884	-1.3902006	-2.463088	-0.08401076	CCNB2
A_33_P3217819	-3.22531	-2.1845481	-0.009819667	-1.1371545	-2.5714529	-0.88201505	CCNE2
A_24_P193592	-4.3723407	-2.2813277	0.078232445	-1.1116413	-2.6840296	-0.5556237	CCNF
A_23_P149200	-8.339797	-3.1441047	-0.005331993	-1.6579813	-2.735531	0.32448134	CDC20
A_23_P70249	-6.2920375	-2.236253	0.0043595633	-1.1567239	-2.5942442	0.05928294	CDC25C
A_23_P385861	-4.4870625	-2.5881345	-0.02719148	-1.3991041	-2.6505241	-0.48475298	CDCA2
A_24_P218979	-6.354238	-3.137648	-0.0060227714	-1.6557064	-2.6368306	0.030888557	CDCA3
A_23_P251421	-8.0611	-2.4415154	0.002456665	-1.2853203	-3.238206	-0.22722912	CDCA7
A_23_P375	-4.631137	-3.1201339	-0.01276048	-1.6543684	-2.7869406	-0.5755742	CDCA8
A_23_P138507	-6.269371	-2.1280746	-0.023222923	-1.1127716	-2.9704316	-0.3221108	CDK1
A_33_P3292540	-5.6947184	-2.7966418	-0.031740505	-1.515436	-2.0784175	0.43120703	CDKN2C
A_23_P48669	-6.6117573	-3.2810607	-7.619858E-4	-1.7149242	-2.8060484	-0.08101463	CDKN3
A_33_P3307903	-5.6388507	-3.3959947	4.5553842E-4	-1.7633787	-2.9419067	-0.44650555	CDKN3
A_24_P413884	-5.0591345	-2.5039837	-0.0049387612	-1.3291639	-2.574661	-0.23577054	CENPA
A_23_P253524	-3.3208454	-2.5399487	-0.031032244	-1.3758316	-2.1929436	-0.46139303	CENPE
A_23_P401	-5.905297	-2.5218418	0.010260582	-1.3242172	-2.750529	-0.18851948	CENPF
A_24_P96780	-3.6587005	-2.312345	-0.007968585	-1.2173252	-2.1404047	-0.26907334	CENPF
A_23_P110802	-6.2891674	-2.3723083	-0.028301239	-1.2745928	-2.7419453	-0.08907636	CENPH
A_24_P462899	-4.63143	-2.6627865	-1.7197926E-4	-1.4131088	-2.4207304	-0.2092727	CENPW
A_23_P115872	-4.829885	-3.2073784	0.0156463	-1.6657482	-2.7095747	-0.43758583	CEP55
A_33_P3291831	-4.393526	-3.5123458	-0.026843706	-1.8392786	-2.3790889	-0.24370956	CEP55
A_33_P3215635	-5.604198	-4.2949443	0.13972981	-1.9629096	-1.2997799	1.1867281	CHRNB2
A_33_P3312301	-4.91786	-2.6805592	-0.02093935	-1.4434733	-1.4697895	0.82824105	CIT
A_23_P420551	-4.4213023	-2.463054	0.059566814	-1.2408813	-2.1994934	-0.055021923	CIT
A_23_P388812	-3.6485562	-2.516003	-0.014812152	-1.3459457	-3.0815842	-1.2142586	CKAP2L

A_23_P117797	-2.2966614	-2.1577103	-0.014031728	-1.1235329	-1.8496599	-0.6501217	CLN6
A_23_P115064	-4.0898514	-2.926105	-0.0071382523	-1.5561198	-3.8269184	-1.7948699	CRABP2
A_23_P429491	-5.6626954	-2.4932537	-0.027401924	-1.3454317	-3.040617	-0.539128	DDIAS
A_23_P200310	-4.000693	-3.1694102	0.026411692	-1.6378027	-2.4417822	-0.44153246	DEPDC1
A_23_P15202	-3.223412	-2.3456335	-0.018756866	-1.2487345	-1.8859185	-0.19732983	DHODH
A_23_P27005	-3.5452936	-2.6375701	0.031163216	-1.3680463	-0.9502204	0.87568474	DHRS11
A_33_P3253960	-2.331859	-2.1663382	0.0059264502	-1.109332	-0.99078685	0.23069382	DHRS11
A_33_P3286218	-3.1575153	-2.5274837	-0.039036434	-1.3767382	-1.6705136	-0.011723836	DLEU2L
A_23_P88331	-5.006406	-3.010574	-0.0687809	-1.6588196	-2.95704	-0.63326484	DLGAP5
A_23_P28953	-2.8636017	-2.3328197	-0.0075149536	-1.2295898	-1.0144278	0.503403	DNMT3B
A_23_P252740	-5.6450586	-2.1492314	-0.044344902	-1.1481658	-3.1510057	-0.65401715	DSCC1
A_23_P96325	-7.5104003	-2.0728397	-0.015694618	-1.0673032	-2.928032	-0.01914215	ERCC6L
A_23_P32707	-6.397801	-2.0636437	0.0059162774	-1.0392777	-1.8796612	0.7979148	ESPL1
A_23_P129221	-3.550998	-2.0680757	-0.0108760195	-1.059165	-1.8133612	0.014863332	FAH
A_33_P3276918	-6.166699	-2.8598409	0.016649246	-1.4992856	-3.1200454	-0.49554715	FAM64A
A_33_P3242952	-6.6121573	-3.6835291	0.013512929	-1.8675756	-2.4852197	0.23990123	FAM72A
A_32_P151800	-7.5542674	-4.2228594	-0.05951913	-2.1377392	-2.3078914	0.60940045	FAM72D
A_23_P323751	-5.4422946	-3.0373344	-0.018978437	-1.6217842	-3.0215816	-0.57736653	FAM83D
A_33_P3257808	-3.8580766	-2.3121192	-0.018641472	-1.2278572	-1.9267474	0.021134377	FANCD2
A_23_P57588	-5.441319	-2.3742805	0.010509491	-1.2369808	-2.526806	-0.08284982	GTSE1
A_24_P38895	-2.7912073	-2.0307229	0.017062187	-1.0049311	-1.4370149	0.043874424	H2AFX
A_23_P393034	-3.4995124	-3.3416188	7.7883404E-4	-1.7397684	-2.5752165	-0.7680626	HAS3
A_23_P349771	-4.5153837	-3.1920803	0.054286797	-1.62021	-1.2130774	0.9617712	HAUS5
A_23_P70448	-5.4957523	-2.4266326	-0.015931448	-1.2948872	-2.5071878	-0.048870724	HIST1H1A
A_33_P3393135	-8.012007	-2.077526	0.044703323	-1.0101633	-3.3642426	-0.362079	HIST1H2AI
A_23_P363174	-4.4900975	-2.0179873	-0.02185885	-1.0347759	-2.888127	-0.72138023	HIST1H2AL
A_23_P431179	-8.415072	-2.02709	-0.06306156	-1.0824718	-1.1706138	1.9023619	HIST1H4A
A_33_P3257678	-13.571829	-2.1178775	-0.09257571	-1.1751947	-2.3766077	1.3859354	HIST2H3A
A_33_P3807062	-5.7132363	-2.6012201	0.010402362	-1.3687862	-2.5380058	-0.023697535	HJURP
A_23_P155765	-6.6508813	-2.3535433	0.0042734146	-1.2305609	-2.1602068	0.5733388	HMGB2
A_32_P1381	-3.2249506	-2.8040144	-0.011084874	-1.4985787	-1.8747686	-0.18549156	HMGN2
A_32_P222383	-3.1351244	-2.7653387	0.01079464	-1.4566616	-1.5919075	0.056615192	HMGN2
A_32_P15799	-2.8218212	-2.5642338	-0.055078823	-1.4136066	-1.5710644	-0.074437775	HMGN2
A_32_P41487	-2.6009188	-2.312202	-0.08683459	-1.2961019	-1.5583191	-0.17929776	HMGN2
A_23_P70007	-5.02233	-3.7079084	0.016185442	-1.8744202	-2.848791	-0.5204341	HMMR
A_24_P123245	-2.6766632	-2.046879	0.003293991	-1.0301319	-1.2973423	0.123093285	HNRNPD
A_23_P105012	-8.164441	-2.0394368	-0.0067594848	-1.0349303	-2.9879522	0.041401863	HRASLS2
A_23_P31945	-11.448499	-11.80173	-0.0050555863	-3.565982	-1.6710973	1.8459892	IL33
A_33_P3321293	-4.741816	-2.7124977	-0.037419003	-1.4770409	-1.7612234	0.48421636	IQGAP3
A_23_P410587	-2.1992805	-2.1517498	-0.0328811	-1.1383915	-1.4294677	-0.29243597	JADE1
A_33_P3334384	-3.321393	-2.3832235	0.004310608	-1.2486037	-1.4316419	0.30014643	KDM4A-AS1
A_23_P127406	-2.3314722	-2.0306258	0.03902356	-0.9829008	-0.9953324	0.22590876	KDM4D
A_24_P227091	-6.1701484	-2.5336382	-5.257924E-4	-1.3417364	-2.4532096	0.17209561	KIF11
A_33_P3230548	-4.337261	-2.2586637	-0.013449351	-1.1889187	-2.292333	-0.17554855	KIF14
A_23_P80902	-8.472745	-2.505511	-0.03958257	-1.3646876	-2.826671	0.2561585	KIF15
A_33_P3242649	-7.639834	-2.4527175	-0.0067739487	-1.3011551	-3.1327515	-0.19921017	KIF18A
A_23_P256956	-8.378264	-3.6935062	0.013962746	-1.8710283	-2.8527954	0.21385606	KIF20A
A_23_P75071	-6.967997	-2.1534045	0.0421079	-1.0645114	-2.6868553	0.11388874	KIF20B
A_33_P3311755	-4.597329	-2.3867922	-0.030905724	-1.2859787	-2.8752632	-0.6744674	KIF23
A_23_P48835	-4.2511163	-2.1520927	0.017656008	-1.0880842	-2.7623959	-0.67455417	KIF23
A_23_P34788	-4.7836237	-2.8770702	-0.0250597	-1.5496601	-2.537958	-0.27985415	KIF2C
A_23_P148475	-6.235765	-2.5076253	0.0029913585	-1.3233305	-2.6702068	-0.029640198	KIF4A
A_33_P3253596	-6.0314097	-2.529894	-0.05321185	-1.3922888	-3.1513546	-0.55885935	KIF4A
A_23_P133956	-7.291927	-2.7344007	0.05792554	-1.3932991	-2.4114938	0.45480633	KIFC1
A_33_P3299761	-2.1956701	-2.6163018	0.02143065	-1.3660984	-2.8391418	-1.7044805	KRT23
A_23_P254081	-2.0507734	-2.2450812	-0.0040512085	-1.1708188	-1.5618706	-0.5257025	LIAS
A_23_P39116	-3.36151	-2.0222394	-0.0071188607	-1.0230726	-1.5177962	0.23131339	LIG1
A_33_P3322400	-6.4940495	-3.4399686	-0.0608414	-1.8432368	-3.0036068	-0.30448833	lnc-TAF1C-1

A_32_P3572	-3.5002673	-3.8125076	-0.015394688	-1.9461349	-2.5726304	-0.7651653	LOC100506797
A_24_P184388	-2.3008583	-2.2051346	-0.021355947	-1.1622227	-1.0803825	0.12178961	LRTOMT
A_23_P92441	-4.8342524	-2.1345446	-0.050928116	-1.1448565	-3.0430415	-0.7697487	MAD2L1
A_24_P346855	-9.419692	-2.925594	-9.988149E-4	-1.5497285	-2.974368	0.26131186	MKI67
A_33_P3374210	-3.5569444	-2.7604442	0.03393491	-1.4309654	-2.5607536	-0.73011523	MKI67
A_23_P3302	-10.994844	-2.3483403	0.0011688868	-1.2304727	-3.2588165	0.19993877	MNS1
A_23_P253752	-4.147857	-2.193383	-0.009339969	-1.1424977	-2.5112264	-0.45886007	MTFR2
A_23_P125705	-2.027995	-2.685083	-0.0033359528	-1.4283026	-0.5672984	0.45275578	NAP1L2
A_33_P3230254	-6.932254	-2.3140118	0.053845722	-1.1565504	-3.3623912	-0.5690667	NCAPG
A_33_P3659876	-5.0625987	-2.0079365	0.021855354	-0.9838584	-2.240167	0.0997111	NCAPG2
A_23_P50108	-5.47819	-2.7579482	-0.003373146	-1.4669685	-2.7042868	-0.25058746	NDC80
A_23_P155711	-8.683721	-2.295577	0.0013620058	-1.1974949	-3.1833951	-0.06508175	NEIL3
A_23_P35219	-5.0534406	-3.1435275	0.045137405	-1.607247	-2.5491035	-0.21183746	NEK2
A_24_P319613	-4.571243	-2.997928	0.025101027	-1.5588646	-2.7242362	-0.5316499	NEK2
A_32_P198731	-7.591552	-2.2209728	-0.017552057	-1.1687437	-3.425455	-0.5010602	NEURL1B
A_23_P69537	-3.4201746	-2.1081367	-0.0015172958	-1.0774857	-2.4359257	-0.6618557	NMU
A_33_P3242863	-10.522606	-3.6451879	0.06913773	-1.7968554	-3.1719952	0.22342491	NT5M
A_33_P3384932	-2.1018968	-2.9574718	-0.00976181	-1.5741262	-0.8083413	0.2633505	NUDT8
A_23_P74349	-5.977636	-3.003399	-0.03322633	-1.6198225	-2.872712	-0.29313692	NUF2
A_33_P3350488	-5.797657	-2.2342846	0.03622532	-1.1235876	-2.4914095	0.04406039	NUSAP1
A_23_P60488	-3.1521425	-2.305783	0.009824435	-1.1954323	-1.0011454	0.6551873	ODF2
A_33_P3308534	-3.5427127	-2.680688	-0.01903995	-1.4416432	-1.8049389	0.01991558	OSBPL1A
A_33_P3358740	-6.5815477	-2.324332	-0.022729238	-1.2395455	-2.1874208	0.53100616	OSBPL7
A_32_P63848	-2.314064	-2.3727136	0.0036881764	-1.2428497	-1.1493851	0.06104374	OXCT1
A_32_P62997	-4.613986	-2.3529665	-0.018670717	-1.2531515	-2.7990904	-0.5930767	PBK
A_23_P30275	-3.5181031	-3.4643762	0.08067354	-1.711922	-1.6067289	0.20806885	PCYOX1L
A_24_P291231	-3.1296449	-2.867004	0.043594677	-1.4759492	-2.2685254	-0.62252635	PER3
A_23_P21436	-3.2614262	-2.1290462	-0.0030787785	-1.0932862	-1.6292583	0.076244675	PHF19
A_23_P21485	-4.6416917	-2.1313615	-0.027681509	-1.1194568	-2.6854799	-0.47082934	PID1
A_23_P416468	-6.4251575	-3.4804745	-0.039446514	-1.8387305	-2.474924	0.20880763	PIF1
A_33_P3628409	-2.2239747	-2.0698328	0.037129402	-1.0123849	-0.47728634	0.675854	PKI55
A_23_P360626	-2.0904105	-2.513408	-0.015625954	-1.3452708	-1.4570583	-0.39327207	PLD6
A_23_P118174	-9.020348	-3.3076637	-0.0092128115	-1.7350254	-2.8380024	0.3351806	PLK1
A_33_P3298387	-3.9145095	-2.4261756	0.011219342	-1.2674646	-2.4067268	-0.4378953	PLK1
A_33_P3243175	-2.2508926	-2.1981027	0.020729383	-1.1155294	-1.2585583	-0.08806101	PNPO
A_33_P3396527	-3.1524744	-2.560399	0.004271189	-1.3520975	-2.2838976	-0.6274131	POLR3G
A_23_P41942	-2.4741232	-2.6548035	-0.029087067	-1.4376922	-2.0426886	-0.7357712	POLR3G
A_23_P206059	-5.768284	-2.3346927	-0.0055742264	-1.2288069	-2.4425793	0.08556303	PRC1
A_33_P3403075	-2.2425382	-2.4419794	-0.00385348	-1.2919044	-1.8714138	-0.70628136	PRR11
A_23_P46539	-8.121693	-5.6058097	-0.0065062842	-2.493429	-2.5394757	0.4823049	PSRC1
A_32_P186474	-2.5859513	-2.3035877	0.01624616	-1.1876364	-2.0785036	-0.7078085	RACGAP1
A_23_P65041	-2.5936937	-2.2508168	-0.0022730827	-1.1727217	-1.9976386	-0.6226304	RACGAP1P
A_33_P3378925	-2.2489223	-2.157795	-0.023166021	-1.1327238	-0.6320222	0.5372117	RBM14
A_23_P141447	-5.8636456	-2.5904603	-0.02323246	-1.396441	-1.8431396	0.7086582	RDM1
A_23_P87351	-2.2668815	-2.1298718	0.03179868	-1.058968	-1.4579312	-0.2772223	RRM1
A_23_P434809	-5.328481	-2.202727	-0.028630257	-1.1679211	-3.3498695	-0.9361451	S100A8
A_33_P3408913	-6.2176204	-3.5796573	0.019081116	-1.8207403	-3.9189522	-1.2825898	SAA2
A_23_P87238	-8.054941	-2.6753576	0.01702404	-1.4027077	-3.9142358	-0.9043617	SAA4
A_24_P3804	-4.6785727	-2.1698513	-0.019132296	-1.1367284	-0.97101563	1.2550529	SAPCD1
A_24_P100517	-4.562731	-3.358742	0.004928271	-1.7429928	-1.9736878	0.21620972	SAPCD2
A_23_P433111	-2.3051093	-2.1535704	0.05573654	-1.0509939	-1.8369331	-0.6320979	SETD9
A_24_P175612	-8.058667	-3.016859	6.041527E-4	-1.5924431	-2.8090134	0.20152791	SFXN2
A_24_P225970	-3.7602174	-2.1305506	-0.013202985	-1.1044294	-2.2223153	-0.31149927	SGOL1
A_23_P411335	-4.9240255	-3.6010122	-0.062848724	-1.9112512	-2.8706818	-0.5708434	SGOL2
A_32_P96719	-5.005011	-2.4701655	-0.013487816	-1.3180956	-2.9013107	-0.5779374	SHCBP1
A_24_P322354	-6.377163	-2.542918	-0.017313639	-1.3637985	-2.9627686	-0.28985372	SKA1
A_23_P89509	-4.189951	-2.1513832	-0.006769816	-1.1120342	-2.3032205	-0.23628712	SPAG5
A_33_P3376116	-9.738439	-2.2100818	-0.0056880317	-1.1497879	-2.8574193	0.4262713	SPC24

A_23_P51085	-5.5927663	-2.5222857	-0.0035476685	-1.3382794	-3.0185916	-0.5350297	SPC25
A_33_P3407400	-2.081119	-2.699632	0.026313782	-1.406449	-0.700165	0.35719457	SPEF2
A_23_P158880	-2.7935076	-2.4612162	-0.0025075276	-1.3018789	-1.3052802	0.17679755	STARD5
A_32_P170444	-2.4733124	-2.259184	-0.034458797	-1.2102604	-1.6566458	-0.35020128	SUB1
A_23_P212844	-3.098872	-2.3954344	0.024062157	-1.2362251	-1.9179233	-0.28618017	TACC3
A_23_P91390	-2.1856909	-2.0912526	0.01916472	-1.0452026	-0.80788106	0.32020822	THBD
A_33_P3210363	-5.401693	-2.4258726	0.030839285	-1.2476645	-2.2989259	0.13448572	TMPO-AS1
A_23_P118834	-7.8589644	-2.7369957	-0.052390736	-1.5049839	-3.392985	-0.41864586	TOP2A
A_23_P68610	-5.158567	-3.0411189	0.00701046	-1.5975918	-2.4666378	-0.09966755	TPX2
A_33_P3339212	-5.3400517	-2.5685387	0.005651156	-1.3552967	-2.466337	-0.0494833	TRIP13
A_33_P3407256	-5.0895085	-2.4029906	-0.008497715	-1.2733287	-2.7201412	-0.37261486	TRIP13
A_33_P3238976	-2.7216873	-2.9059985	-0.024021467	-1.5630554	-1.6139412	-0.16943996	TRMT5
A_23_P150935	-5.968365	-2.6703787	0.0065755844	-1.4104687	-2.3057196	0.2716163	TROAP
A_23_P259586	-6.2865787	-2.3098094	-0.0037234623	-1.2114973	-2.8347816	-0.18250656	TTK
A_24_P297539	-4.6971574	-3.2715468	-2.1966298E-4	-1.7101927	-2.0037959	0.22799206	UBE2C
A_23_P40989	-4.00768	-2.2221513	-0.0011622111	-1.1531192	-1.8154625	0.18730481	USP13
A_33_P3341499	-6.0192633	-2.0449362	-0.009203593	-1.0412593	-1.5355641	1.0540229	WNT5A
A_23_P53363	-2.393303	-2.357703	0.010797501	-1.2265844	-1.6257181	-0.3667151	XRCC6BP1
A_33_P3238171	-2.4515333	-2.2901173	0.007037481	-1.1883839	-0.95311755	0.34056678	ZDHHC8
A_33_P3423420	-6.8878775	-3.0357807	0.022458553	-1.579609	-4.2826324	-1.4985727	ZNF750
A_33_P3333187	-9.274679	-3.7844915	0.0019108454	-1.9181887	-2.9094353	0.3038621	
A_32_P152696	-4.1945944	-3.3422978	-0.001124382	-1.7419647	-1.9586855	0.109845795	
A_33_P3349840	-3.963979	-2.9122066	-0.032503765	-1.5746164	-1.9458097	0.041139603	
A_33_P3263157	-3.7493498	-2.8114715	-0.031777065	-1.5231024	-1.8851261	0.021514257	
A_33_P3259902	-2.994078	-3.163065	-0.022871336	-1.6841946	-1.7203192	-0.13820744	
A_33_P3301394	-2.8098104	-3.030808	-0.0656929	-1.6653954	-1.7149906	-0.22451782	
A_23_P388146	-2.787285	-2.341825	0.012421926	-1.2152113	-0.95854187	0.5203187	

Table S2. List of genes downregulated in OIS and after plasma membrane depolarization.

Gene Name1	E2Fs ChIP-seq positive2	FC expression3
**Down-regulated GO mitosis**		
ASPM	4	0.36
AURKA	4	0.36
CCNA2	5	0.27
CCNB1	3	0.33
CCNB2	3	0.38
CDC20	4	0.32
CDCA2	4	0.38
CDCA3	4	0.32
CDCA8	7	0.32
CENPW	5	0.38
CEP55	6	0.28
CIT	5	0.37
DLGAP5	5	0.32
FAM64A	4	0.35
FAM83D	5	0.32
HAUS5	4	0.33
KIF2C	5	0.34
KIF4A	7	0.38
KIFC1	7	0.38
MKI67	5	0.34
NDC80	5	0.36
NEK2	5	0.33
NUF2	5	0.33
PLK1	5	0.30
PSRC1	0	0.18
RECQL5	2	0.31
SGOL2	4	0.27
TOP2A	4	0.35
TPX2	5	0.33
TUBB3	2	0.32
UBE2C	4	0.31
**Up-regulated**		
ACSS3	0	13.95
ANK1	0	58.57
AQP3	0	12.60
ATP8A2	1	13.37
CA8	0	50.19
CALB1	1	12.01
CAMK2B	0	10.83
CAPN8	0	17.78
CAPN8	0	13.48
CPO	0	10.72
DENND2D	1	11.09

DMBT1	0	16.05
FAM43B	0	11.97
FXYD2	0	22.99
GPER1	1	10.44
ISYNA1	0	15.48
MYH15	0	20.02
PLCXD3	0	19.43
PRSS35	0	43.58
RARRES2	0	16.81
SHISA2	0	23.50
SLC38A4	0	9.79
SLC7A7	0	12.26
SNX31	0	12.99
SPTSSB	1	10.03
ST8SIA1	0	26.09
SYT12	0	11.35
TNFSF15	0	15.27
UCA1	0	13.66
WIPF1	0	9.85
**Down-regulated**		
ADAMTS9	0	0.28
APOBEC3A	0	0.14
ARHGAP11A	1	0.35
BCL2A1	0	0.14
C8orf89	0	0.21
CHRNB2	0	0.26
GBP6	0	0.11
HMSD	0	0.26
IVL	0	0.22
KANK4	0	0.24
KRT34	0	0.20
LCE2A	0	0.26
LCE2C	0	0.28
MAL2	3	0.27
MGAM	0	0.26
MMP10	0	0.28
NPNT	1	0.20
NRADDP	0	0.14
NT5M	3	0.29
PPBP	0	0.29
RS1	0	0.13
RTKN2	3	0.29
SAA2	0	0.29
SEMA5B	0	0.20
SERPINB2	0	0.16
SPINK6	0	0.27

SPOCD1	0	0.23
SPRR2D	0	0.25
SPRR2G	0	0.25
TMCC3	0	0.27
**GO mitosis not down**		
AKAP8	0	1.03
ANKRD53	0	1.04
ARHGEF10	1	1.17
ATM	2	0.95
ATRX	2	1.71
AURKC	0	1.51
BECN1	0	0.93
BIRC7	0	1.11
BMP4	0	3.18
BMP7	0	0.91
BOD1	2	0.91
BUB3	2	0.57
CCDC8	0	1.08
CD28	0	1.12
CDC14A	1	0.98
CDC16	1	0.96
CDK11A	0	1.24
CDK13	2	1.20
CHMP5	2	1.03
CLTC	1	0.93
CUL3	2	1.16
DAPK3	0	1.31
EPGN	0	0.54
EPS8	1	1.15
EREG	0	2.16
FSD1	0	2.19
INS	0	0.86
NPM2	0	1.44
NSMCE2	1	1.24
NEK3	0	0.90

1 Top 30 genes for each class of regulated genes in response to KCL exposure in HEC cells
2 Number of ChIP-seq experiments showing binding of E2F1 and E2F4 transcription factors for each genes included in the table according to Encode database (Peaks with score above 500).
3 Fold Change expression data from microarray expreriment we have done on HEC cells exposed to KCL during 24h.

Table S3. Average number of ChIP-seq experiments showing E2F binding sites.
